# Synthesis of triphenylene-fused phosphole oxides via C–H functionalizations

**DOI:** 10.3762/bjoc.16.48

**Published:** 2020-03-27

**Authors:** Md Shafiqur Rahman, Naohiko Yoshikai

**Affiliations:** 1 Division of Chemistry and Biological Chemistry, School of Physical and Mathematical Sciences, Nanyang Technological University, Singapore 637371, Singapore

**Keywords:** C–H functionalization, fluorescence, phosphole, polycyclic aromatic hydrocarbons, triphenylene

## Abstract

The synthesis of triphenylene-fused phosphole oxides has been achieved through two distinct C–H functionalization reactions as key steps. The phosphole ring was constructed by a three-component coupling of 3-(methoxymethoxy)phenylzinc chloride, an alkyne, and dichlorophenylphosphine, involving the regioselective C–H activation of the C2 position of the arylzinc intermediate via 1,4-cobalt migration. The resulting 7-hydroxybenzo[*b*]phosphole derivative was used for further π-extension through Suzuki–Miyaura couplings and a Scholl reaction, the latter closing the triphenylene ring. The absorption and emission spectra of the thus-synthesized compounds illustrated their nature as hybrids of triphenylene and benzo[*b*]phosphole.

## Introduction

**Figure 1:**
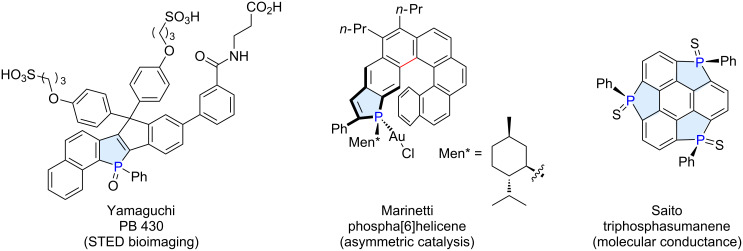
Examples of functional molecules based on π-extended phospholes.

The phosphorus-containing five-membered ring, phosphole, has attracted significant attention as a structural motif in π-conjugated functional molecules [1–9]. Its inherently unique electronic structure, along with opportunities to modify the phosphorus center and the periphery by substitution or ring fusion, have stimulated chemists to explore a structurally diverse range of phosphole derivatives with extended π-system. These included, in particular, those fused with polycyclic aromatic hydrocarbons (PAHs) for possible applications in organic electronics, bioimaging and sensing, and asymmetric catalysis (Figure 1). To name a few examples, Yamaguchi et al. described synthetic routes to novel phosphorus-containing ladder molecules and their application as fluorescence probes for biological imaging [10–11]. Marinetti extensively studied the synthesis of phosphahelicenes with linear fusion of the phosphole and the carbohelicene units [12], and their applications in asymmetric catalysis [13] and organic light-emitting diodes [14]. Recently, Saito et al. reported the synthesis of phosphorus-bridged triphenylenes, that is, triphosphasumanene trisulfides, and demonstrated their capability as a junction for single-molecule conductors [15].

**Scheme 1:**
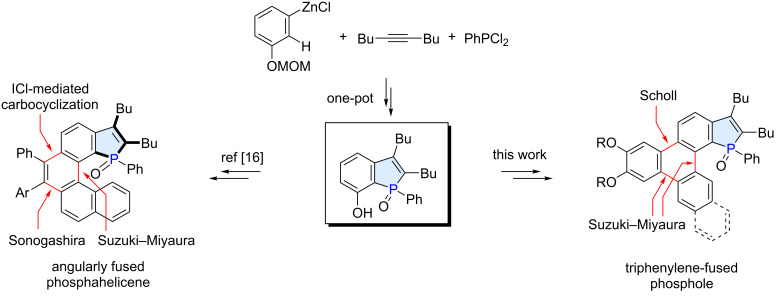
Syntheses of PAH-fused phospholes featuring a 7-hydroxybenzo[*b*]phosphole as a key intermediate.

The synthesis of PAH-fused phospholes typically requires efficient methods for the construction of the phosphole ring as well as for the π-extension/fusion of the PAH moiety, which should be smoothly implemented into the overall synthetic planning. In this context, we have recently reported the synthesis of novel phosphahelicenes that featured angular fusion of the phosphole and the carbohelicene moieties (Scheme 1) [16]. The approach focused on the regioselective one-pot synthesis of a 7-hydroxybenzo[*b*]phosphole derivative from an 3-alkoxyphenylzinc reagent, an alkyne, and dichlorophenylphosphine [17]. The hydroxy group of this key intermediate served as a handle for the π-extension through a Suzuki–Miyaura coupling, Sonogashira coupling, and electrophilic alkyne carbocyclization [18].

Given the successful synthesis of the angularly fused phosphahelicenes, we became interested in the further exploitation of 7-hydroxybenzo[*b*]phosphole as an intermediate for the synthesis of π-extended phospholes. In this respect, our attention focused on the fusion of phosphole with triphenylene, which represents one of the most common disc-like PAH motifs in organic materials chemistry [19–25]. Herein, we report on the synthesis of triphenylene-fused phosphole oxides, which are distinct from Saito’s compounds [15] as well as from other reported examples [26–28] in terms of the mode of fusion of the phosphole and triphenylene units. The present phosphole/triphenylene hybrid molecules displayed absorption and emission profiles that reflected the characteristics of both triphenylene and benzo[*b*]phosphole.

## Results and Discussion

**Scheme 2:**
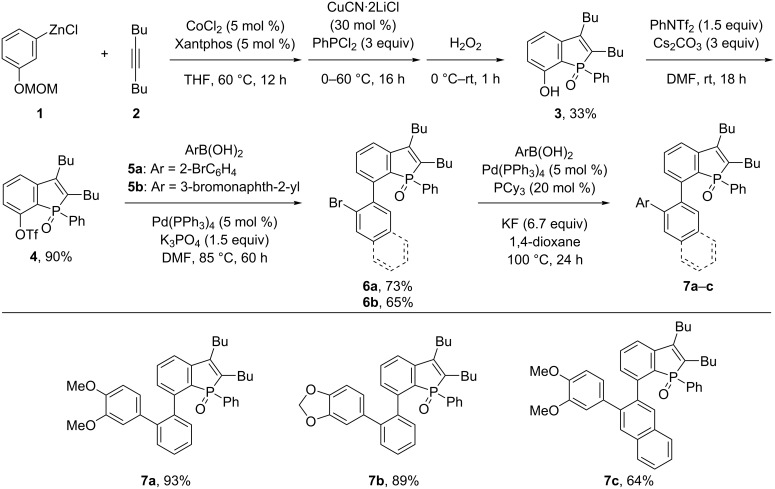
Synthesis of phosphole-fused *ortho*-teraryl compounds **7**.

The present synthetic study commenced with the recently reported preparation of 7-hydroxybenzo[*b*]phosphole derivative **3** from 3-(methoxymethoxy)phenylzinc (**1**), 5-decyne (**2**), and PhPCl_2_ in the presence of a cobalt–diphosphine catalyst (Scheme 2). This one-pot construction of the benzo[*b*]phosphole core ensured the preferential phosphole ring closure in proximity of the alkoxy group of the arylzinc reagent **1** (regioselectivity of ≈3:1), presumably due to a secondary interaction between the MOM group and the cobalt catalyst during the key C–H activation step, i.e., 1,4-cobalt migration in the alkenylcobalt intermediate [29]. The oxidation of the benzo[*b*]phosphole phosphorous atom and cleavage of the MOM group took place simultaneously, and thus afforded compound **3** in 33% yield on a 5 mmol scale [16]. Compound **3** was then converted to the triflate, and subjected to Suzuki–Miyaura couplings with 2-bromophenylboronic acid (**5a**) or 3-bromonaphth-2-ylboronic acid (**5b**) to afford the phosphole-fused biaryls **6a** and **6b**, respectively, in decent yields. Subsequent Suzuki–Miyaura couplings of **6a** or **6b** with 3,4-dialkoxyarylboronic acids furnished the phosphole-fused *ortho*-teraryl products **7a**–**c** in moderate to high yields.

**Scheme 3:**
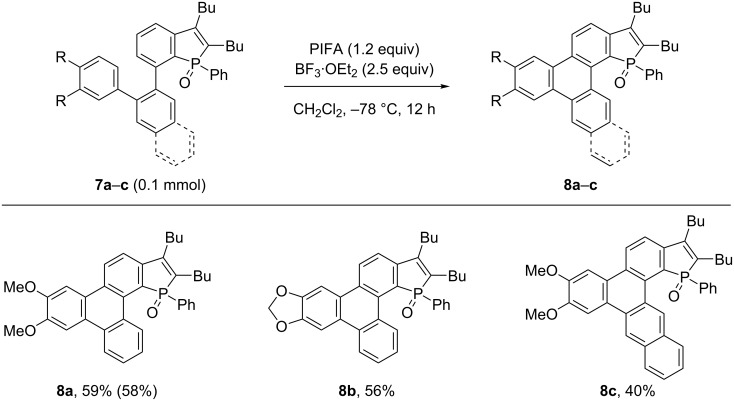
Oxidative cyclization of phosphole-fused *ortho*-teraryl compounds **7** into triphenylene-fused phosphole oxides **8**. The yields in parentheses were obtained in a 0.5 mmol-scale reaction.

With the phosphole-fused *ortho*-teraryl compounds **7** in hand, we next examined their cyclization into triphenylene derivatives by the Scholl reaction (Scheme 3) [30]. The reaction of **7a** (0.1 mmol) in the presence of [bis(trifluoroacetoxy)iodo]benzene] (PIFA) and BF_3_·OEt_2_ in dichloromethane at −78 °C afforded, after 12 h, the desired cyclized product **8a** in 59% yield. The reaction could be performed on a 0.5 mmol scale in a similar yield of 58%. Note that other typical reagents used for the Scholl reaction, such as DDQ/CF_3_CO_2_H, FeCl_3_, Cu(OTf)_2_, and AlCl_3_ failed to promote the cyclization of **7a** to **8a**. The PIFA/BF_3_·OEt_2_ system also promoted the Scholl reaction of terphenyl **7b** bearing a methylenedioxy moiety with a comparable efficiency to afford **8b** in 56% yield. Compound **8c**, a naphthylene-linked analogue of **8a**, also underwent cyclization under the same conditions to give the corresponding product **8c** albeit in a somewhat lower yield of 40%.

**Figure 2:**
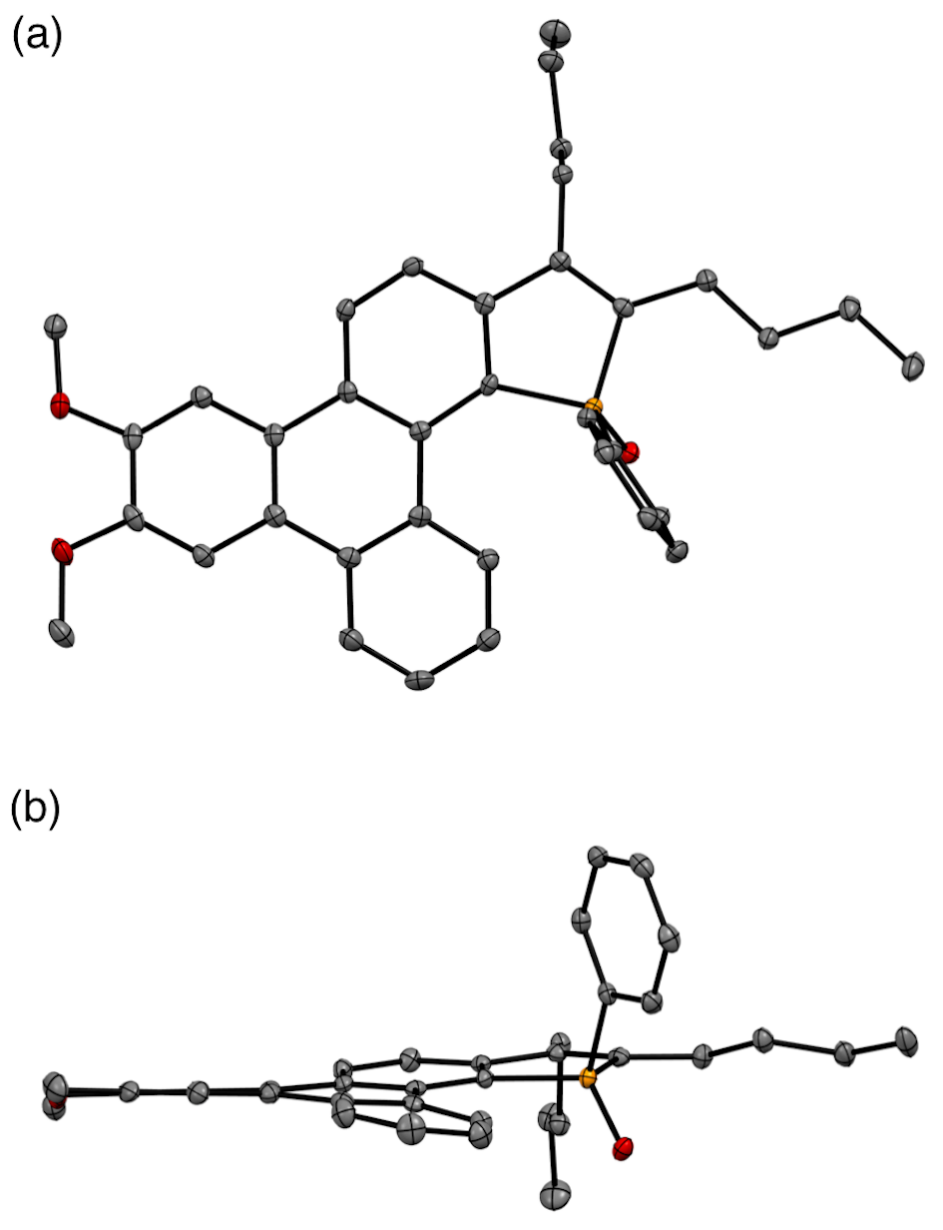
ORTEP drawings of compound **8a** (thermal ellipsoids set at 50% probability). a) top view; b) side view).

The triphenylene-fused phosphole oxide **8a** was recrystallized from CH_2_Cl_2_, and the molecular structure was unambiguously confirmed by single crystal X-ray analysis (Figure 2) [31]. As can be seen from the side view, the triphenylene moiety slightly deviated from planarity, because the fusion with the phosphole ring caused a subtle steric repulsion between the phosphorus substituents and the triphenylene edge within the phospha[4]helicene moiety. Unlike many triphenylene derivatives, the crystal packing of **8a** did not involve columnar π–π stacking of the PAH moiety (Figure S1, Supporting Information File 1). This is likely due to the fact that such π-stacking is inhibited by the steric bulk of the phosphole substituents (i.e., the butyl groups, the phenyl group, and the oxygen atom).

**Figure 3:**
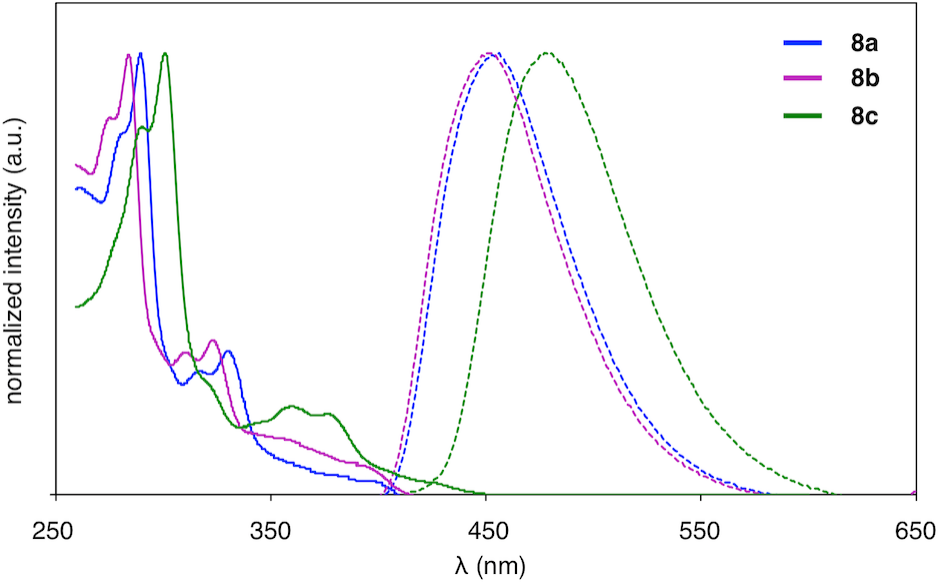
UV–vis absorption (solid lines) and fluorescence (dashed lines) spectra of compounds **8a**–**c**.

Upon the successful synthesis of the triphenylene-fused phosphole oxides **8**, we studied the absorption and emission properties of these compounds in CH_2_Cl_2_ solution. Figure 3 shows the absorption and emission spectra of compounds **8a**–**c**, and Table 1 provides a summary of these spectra and reported spectral data of structurally related benzo[*b*]phosphole and triphenylene derivatives. The optical data illustrate the nature of compounds **8a**–**c** as hybrids of triphenylene and benzo[*b*]phosphole oxide. Regarding the absorption, **8a** and **8b** displayed multiple absorption bands from 250–400 nm (Table 1, entries 1 and 2), which reflected the characteristics of PAHs including triphenylene derivatives (Table 1, entries 5 and 6) [32–34], rather than the 2,3-dialkylbenzo[*b*]phosphole core (Table 1, entry 4). As expected, the absorption of **8c** showed a bathochromic shift compared to **8a** and **8b** as a result of π-extension (Table 1, entry 3). Like many 2,3-dialkylbenzo[*b*]phospholes [17,35], **8a**–**c** showed strong fluorescence in solution (Φ_F_ = 0.67 and 0.34 for **8a** and **8b**, respectively). In contrast to the highly resolved bands in the absorption spectra, the fluorescence spectra were rather simple, with distinct emission peaks at 452 nm (**8a**), 450 nm (**8b**), and 477 nm (**8c**). Such a behavior was distinct from the fluorescence of the parent triphenylene and 2,3-dialkoxytriphenylene, which have been reported to show multiple emission peaks (Table 1, entries 5 and 6).

**Table 1:** Summary of the absorption and emission spectra.^a^

entry	compound	λ_abs_ (nm)^b^	log ε_max_^c^	λ_em_ (nm)	Φ_F_^d^

1	**8a**	289, 330, 393 (sh)	4.16	452	0.67
2	**8b**	284, 324, 387 (sh)	4.27	450	0.34
3	**8c**	301, 361, 420 (sh)	4.41	477	–
4^e^	BP	320	3.27	387	0.11
5^f^	TP	260, 285, 320 (sh)	4.23	355, 364, 371	0.02
6^g^	TP(OC_12_H_25_)_2_	277, 298, 356	–	364, 382	–

^a^Measured in CH_2_Cl_2_ at 5 × 10^−6^ M. ^b^Representative absorption maxima (sh stands for a shoulder peak). ^c^Molar absorption coefficient for the longest-wavelength absorption maximum (except the shoulder). ^d^Determined using quinine sulfate as the standard (54% in 0.1 M H_2_SO_4_). ^e^BP = 1-phenyl-2,3-dibutylbenzo[*b*]phosphole oxide. Data taken from [17]. ^f^TP = triphenylene. Data taken from [32] (λ_abs_ and log ε) and [33] (λ_em_ and Φ_F_). ^g^TP(OC_12_H_25_)_2_ = 2,3-di(*n*-dodecyloxy)triphenylene. Data taken from [34].

## Conclusion

In summary, we synthesized novel triphenylene-fused phosphole oxides through C–H functionalization and cross-coupling reactions. The phosphole ring was constructed in the early stage of the synthesis by a three-component assembly method featuring a 1,4-cobalt migration as the key step. Unlike other C–H activation/alkyne annulation approaches to benzo[*b*]phospholes [36–40], this three-component method guarantees a good regioselectivity for the formation of the desired 7-hydroxybenzo[*b*]phosphole derivatives. The triphenylene moiety was completed in the last step through a Scholl reaction. The synthesized triphenylene-fused phosphole oxides showed strong blue fluorescence in solution. The absorption and emission profiles of the π-extended phosphole oxide revealed their characteristics as hybrids of 2,3-dialkoxytriphenylene and 1-phenyl-2,3-dialkylbenzo[*b*]phosphole. We anticipate that the key intermediate of the present synthesis, **3**, and related benzo[*b*]phospholes accessible by the three-component assembly hold promise for further explorations inot novel π-extended phosphole derivatives.

## Supporting Information

File 1 Experimental details and characterization data of new compounds.

File 2 Crystallographic data for compound **8a**.
