# Supplementary material for: Synthesis of triphenylene-fused phosphole oxides via C–H functionalizations
Source: Beilstein J Org Chem. 2020 Mar 27;16:524–9. doi: 10.3762/bjoc.16.48 (PMC7113549; doi:10.3762/bjoc.16.48)

BEILSTEIN JOURNAL OF ORGANIC CHEMISTRY

Supporting Information

for

Synthesis of triphenylene-fused phosphole oxides via C–H functionalizations

Md. Shafiqur Rahman and Naohiko Yoshikai

Beilstein J. Org. Chem. **2020,** *16,* 524–529. doi:10.3762/bjoc.16.48

Experimental details and characterization data of new compounds

License and Terms: This is a supporting information file under the terms of the Creative Commons Attribution License (http://creativecommons.org/licenses/by/4.0). Please note that the reuse, redistribution and reproduction in particular requires that the authors and source are credited.
The license is subject to the *Beilstein Journal of Organic Chemistry* terms and conditions: (https://www.beilstein-journals.org/bjoc)

Table of contents

Materials and methods S2

Synthesis of triphenylene-fused phosphole oxides S3

Copies NMR spectra S11

Materials and methods

General. All reactions with air or moisture-sensitive compounds were performed using standard Schlenk techniques in oven-dried reaction vessels under a nitrogen or argon atmosphere. Analytical thin-layer chromatography (TLC) was performed on Merck 60 F254 silica gel plates. Flash column chromatography was performed using 40–63 μm silica gel (Si 60, Merck). ^{1}H, ^{13}C and ^{31}P nuclear magnetic resonance (NMR) spectra were recorded on JEOL ECA-400 (400 MHz) or Bruker AV-400 (400 MHz) and AV-300 (300 MHz) NMR spectrometers. ^{1}H and ^{13}C NMR spectra are reported in parts per million (ppm) downfield from an internal standard, tetramethylsilane (0 ppm) and $CHCl_3$ (77.0 ppm), respectively. ^{31}P NMR spectra are referenced to an external reference (85% H_3PO_4, 0 ppm). High-resolution mass spectra (HRMS) were obtained with a Q-Tof Premier LC HR mass spectrometer. Melting points were determined using a capillary melting point apparatus and are uncorrected. UV–vis and fluorescence spectra were recorded on a Shimadzu UV-1800 spectrophotometer and a Shimadzu RF-5301PC spectrofluorophotometer, respectively.

Materials. Unless otherwise noted, commercial reagents were purchased from Alfa Aesar, Sigma Aldrich, TCI and other commercial suppliers and used as received. Xantphos and $CoCl_2$ (anhydrous, 97%) were purchased from Alfa Aesar and used as received. Tetrahydrofuran (THF) and 1,4-dioxane were distilled over Na/benzophenone. Dichloromethane (DCM) was distilled over calcium hydride. Anhydrous dimethylformamide (DMF) was purchased from Sigma-Aldrich. 3-(Methoxymethoxy)phenylmagnesium bromide was prepared from the corresponding 3-(methoxymethoxy)phenyl bromide and magnesium turnings in anhydrous THF and titrated before use.

Synthesis of triphenylene-fused phosphole oxides

The synthesis and characterization of 7-hydroxybenzo[*b*]phosphole oxide (**3**) and its triflate **4** are described in our previous work.[1]

Suzuki–Miyaura coupling between 4 and bromoarylboronic acid

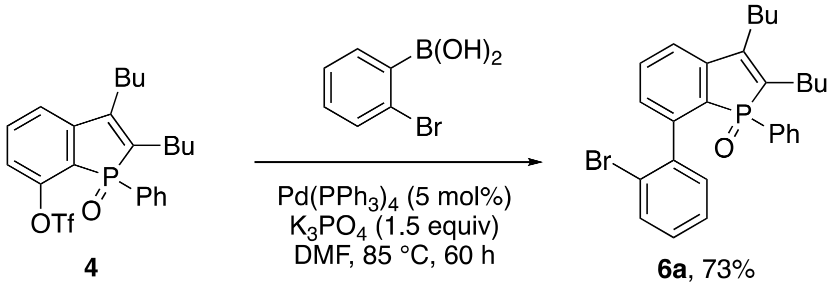

Typical procedure: 7-(2-Bromophenyl)-2,3-dibutyl-1-phenylphosphindole 1-oxide (6a). A 25 mL oven-dried Schlenk tube was charged with **4** (0.97 g, 2.0 mmol) and DMF (6 mL). The resulting solution was degassed through freeze-pump-thaw cycles (3 times). To the degassed solution, $Pd(PPh_3)_4$ (115.6 mg, 0.10 mmol), K_3PO_4 (0.64 g, 3.0 mmol), and 2-bromophenylboronic acid (0.48 g, 2.4 mmol) were added sequentially. The resulting mixture was stirred at 85 °C for 60 h and then allowed to cool to room temperature. The reaction mixture was extracted with EtOAc (3 × 30 mL). The combined organic layer was dried over $MgSO_4$ and concentrated under reduced pressure. The residue was purified by flash chromatography on silica gel (eluent: EtOAc/hexane 1:2) to afford the title compound as colorless oil (0.72 g, 73%). Signal broadening was observed in the ^{1}H and ^{13}C NMR spectra of this compound and compound **7b**, presumably due to the slow rotation of the aryl–aryl bond. The presence of a minor peak in the corresponding ^{31}P NMR spectra suggests the existence of a mixture of equilibrating diastereomers.

^{1}H NMR (400 MHz, $CDCl_3$, 298 K): δ 7.68 (d, J = 7.7 Hz, 1H), 7.49 (t, J = 7.7 Hz, 1H), 7.42-7.35 (m, 3H), 7.28 (d, J = 8.2 Hz, 1H), 7.19-7.11 (m, 6H), 2.66 (t, J = 8.0 Hz, 2H), 2.56-2.44 (m, 1H), 2.32-2.21 (m, 1H), 1.67-1.60 (m, 2H), 1.57-1.48 (m, 2H), 1.45-1.36 (m, 2H), 1.31-1.25 (m, 2H), 1.02 (t, J = 7.2 Hz, 3H), 0.79 (t, J = 7.6 Hz, 3H); ^{13}C NMR (75 MHz, $CDCl_3$, 298 K): δ 150.8 (d, J_{PC} = 19.5 Hz), 143.6 (d, J_{PC} = 28.4 Hz), 143.2 (d, J_{PC} = 7.9 Hz), 138.2 (2C), 134.3, 133.7 (d, J_{PC} = 98.6 Hz), 132.2 (2C), 131.8, 131.5, 131.0 (d, J_{PC} = 95.1 Hz), 130.5, 130.2, 128.7 (d, J_{PC} = 92.5 Hz), 128.3, 127.9, 126.7, 122.6, 120.6 (d, J_{PC} = 10.7 Hz), 30.8 (d, J_{PC} = 1.6 Hz),

30.7 (d, J_{PC} = 1.1 Hz), 26.4 (d, J_{PC} = 12.9 Hz), 26.1 (d, J_{PC} = 11.0 Hz), 23.2, 22.9, 14.0, 13.7; ^{31}P NMR (161 MHz, $CDCl_3$, 298 K): δ 39.5 (major), 37.2 (minor); HRMS (ESI) Calcd for $C_{28}H_{31}BrOP$ [M + H]$^+$ 493.1296, found 493.1286.

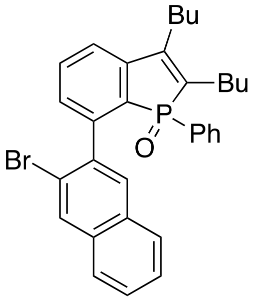

7-(3-Bromonaphthalen-2-yl)-2,3-dibutyl-1-phenylphosphindole 1-oxide (6b): The reaction was performed on a 0.5 mmol scale according to the typical procedure to afford the title compound as colorless oil (182.6 mg, 65%).

^{1}H NMR (400 MHz, $CDCl_3$, 298 K): δ 8.22 (s, 1H), 7.98 (app. doublet, *J* = 7.5 Hz, 1H), 7.80 (s, 1H), 7.69 (app. doublet, *J* = 7.2 Hz, 1H), 7.52 (t, *J* = 6.6 Hz, 3H), 7.40 (dd, *J* = 7.6, 2.3 Hz, 1H), 7.31 (app. d, *J* = 6.9 Hz, 2H), 7.05-6.98 (m, 4H), 2.68 (t, *J* = 7.8 Hz, 2H), 2.54-2.45 (m, 1H), 2.30-2.17 (m, 1H), 1.70-1.66 (m, 2H), 1.58-1.49 (m, 2H), 1.44-1.38 (m, 2H), 1.29-1.19 (m, 2H), 1.03 (t, *J* = 7.3 Hz, 3H), 0.78 (t, *J* = 7.2 Hz, 3H); ^{13}C NMR (100 MHz, $CDCl_3$, 298 K): δ 150.8 (d, J_{PC} = 18.0 Hz), 143.7 (d, J_{PC} = 28.8 Hz), 143.1 (d, J_{PC} = 7.9 Hz), 135.0, 134.8 (d, J_{PC} = 115.0 Hz), 133.8, 133.3, 132.7 (overlapped, 2C), 131.7 (d, J_{PC} = 102.1 Hz), 131.6, 131.5, 131.0 (d, J_{PC} = 11.1 Hz), 130.7, 130.4, 130.3, 128.9, 128.8, 128.1 (overlapped, 2C), 127.7 (d, J_{PC} = 102.5 Hz), 126.4 (d, J_{PC} = 35.6 Hz), 120.6 (d, J_{PC} = 10.9 Hz), 120.1, 30.9, 30.7, 26.5 (d, J_{PC} = 13.0 Hz), 26.1 (d, J_{PC} = 10.6 Hz), 23.2, 23.0, 14.0, 13.7; ^{31}P NMR (161 MHz, $CDCl_3$, 298 K): δ 39.8 (major), 37.5 (minor); HRMS (ESI) Calcd for $C_{32}H_{33}BrOP$ [M + H]$^+$ 543.1452, found 543.1463.

Suzuki–Miyaura coupling between 6a/6b and arylboronic acid

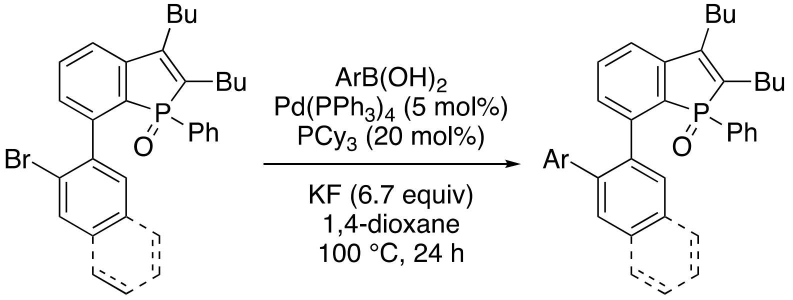

6a or **6b** **7a–7c**

Typical procedure: In a 25 mL Schlenk tube, **6a** or **6b** (0.3 mmol) was dissolved in 1,4-dioxane (2.0 mL) under a N_2 atmosphere. Then, $Pd_2(dba)_3$ (8.3 mg, 9.0 µmol), PCy_3 (16.9 mg, 0.060 mmol), KF (116.2 mg, 2.0 mmol), and arylboronic acid (0.36 mmol) were added sequentially. The resulting mixture was stirred at 100 °C for 24 h. The reaction mixture was allowed to cool to room temperature, diluted with water, and extracted with EtOAc (3 × 20 mL). The combined organic layer was dried over $MgSO_4$ and concentrated under reduced pressure. The residue was purified by flash chromatography on silica gel (eluent: EtOAc/hexane 1:2) to afford the desired *ortho*-teraryl product.

Note: The ^{1}H NMR spectra of teraryl compounds **7a–c** ($CDCl_3$, 298 K) displayed extensive signal broadening, and most of the signals did not show resolved spin–spin coupling. Some of the ^{13}C NMR signals also showed substantial broadening, which made it difficult to match the number of the observed peaks to the number of the unique carbons. This is presumably due to the restricted, slow rotation of the aryl–aryl bonds compared to the time scale of the NMR measurement.

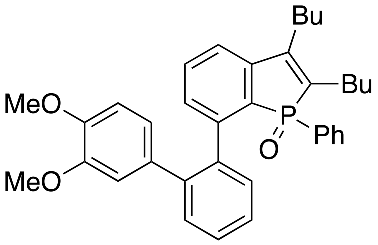

2,3-Dibutyl-7-(3',4'-dimethoxy-[1,1'-biphenyl]-2-yl)-1-phenylphosphindole 1-oxide (7a): The reaction was performed on 1.0 mmol scale, and the title compound was obtained as colorless

oil (0.51 g, 93%). ^{1}H NMR (400 MHz, $CDCl_3$, 298 K): δ 8.04 (brs, 1H), 7.52-7.36 (m, 5H), 7.19-7.12 (m, 5H), 6.73-6.57 (m, 2H), 5.96 (brs, 1H), 5.66 (brs, 1H), 3.79 (s, 3H), 3.35 (s, 3H), 2.67 (app. brs, 3H), 2.33-2.31 (m, 1H), 1.77-1.59 (m, 3H), 1.56-1.47 (m, 3H), 1.42-1.25 (m, 2H), 1.02 (t, J = 7.2 Hz, 3H), 0.83 (app. triplet, J = 7.2 Hz, 3H); ^{13}C NMR (100 MHz, $CDCl_3$, 298 K): δ 147.7 (d, J_{PC} = 30.3 Hz), 144.5 (d, J_{PC} = 8.1 Hz), 133.7, 133.4, 131.8, 131.3, 131.0 (d, J_{PC} = 107.3 Hz), 130.5, 129.0 (d, J_{PC} = 124.3 Hz), 128.5, 121.7, 119.7 (d, J_{PC} = 11.1 Hz), 112.8, 110.3, 55.8, 55.7, 31.0, 30.9 (d, J_{PC} = 1.5 Hz), 26.5, 26.4, 23.2, 23.1, 14.0, 13.7 (Note: The number of well-resolved ^{13}C peaks in the downfield region (> 100 ppm; 14) was less than the number of unique aromatic carbons (24) due to extensive signal broadening); ^{31}P NMR (161 MHz, $CDCl_3$, 298 K): δ 40.3 (major), 38.2 (minor); HRMS (ESI) Calcd for $C_{36}H_{40}O_3P$ $[M + H]^+$ 551.2715, found 551.2709.

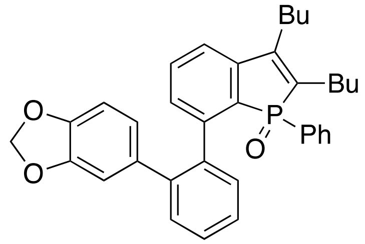

7-(2-(Benzo[*d*][1,3]dioxol-5-yl)phenyl)-2,3-dibutyl-1-phenylphosphindole 1-oxide (7b): The reaction was performed on 0.3 mmol scale. The title compound was obtained as colorless oil (143 mg, 89%). ^{1}H NMR (400 MHz, $CDCl_3$, 298 K): δ 7.95 (brs, 1H), 7.52-7.32 (m, 4H), 7.20-7.12 (m, 6H), 6.62 (dd, J = 7.3, 2.3 Hz, 1H), 6.40 (brs, 1H), 5.82 (s, 2H), 5.64 (app. singlet, 1H), 5.56 (app. singlet, 1H), 2.67 (t, J = 5.5 Hz, 2H), 2.61-2.57 (m, 1H), 2.34 (app. brs, 1H), 1.75-1.61 (m, 3H), 1.57-1.49 (m, 3H), 1.39-1.26 (m, 2H), 1.02 (t, J = 7.2 Hz, 3H), 0.83 (t, J = 7.3 Hz, 3H); ^{13}C NMR (100 MHz, $CDCl_3$, 298 K): δ 146.4 (d, J_{PC} = 83.1 Hz), 144.3 (d, J_{PC} = 8.1 Hz), 140.1, 136.5, 135.1, 131.7, 131.4 (d, J_{PC} = 101.4 Hz), 131.3, 131.2 (d, J_{PC} = 112.0 Hz), 130.9 (d, J_{PC} = 84.1 Hz), 129.7, 128.4 (d, J_{PC} = 12.1 Hz), 128.2, 126.6 (d, J_{PC} = 4.1 Hz), 122.8, 119.7 (d, J_{PC} = 10.8 Hz), 109.7, 107.4, 100.7, 31.0 (d, J_{PC} = 1.6 Hz), 30.8 (d, J_{PC} = 1.6 Hz), 26.5 (d, J_{PC} = 13.2 Hz), 26.2 (d, J_{PC} = 10.7 Hz), 23.2, 23.1, 14.0, 13.7 (Note: The number of well-resolved ^{13}C peaks in the downfield region (> 100 ppm; 19) was less than the number of unique aromatic/ether carbons (25) due to extensive signal broadening); ^{31}P NMR (161 MHz, $CDCl_3$, 298 K): δ 39.9; HRMS (ESI) Calcd for $C_{35}H_{36}O_3P$ $[M + H]^+$ 535.2402, found 535.2399.

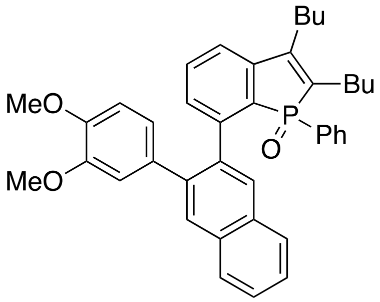

2,3-Dibutyl-7-(3-(3,4-dimethoxyphenyl)naphthalen-2-yl)-1-phenylphosphindole 1-oxide (7c): The reaction was performed on 0.3 mmol scale. The title compound was obtained as colorless oil (115 mg, 64%). The observation of two ^{31}P NMR signals suggested that the compound exists as a mixture of slow-equilibrating diastereomers. ^{1}H NMR (400 MHz, $CDCl_3$, 298 K): δ 8.6 (app. brs, 1H), 7.84-7.72 (m, 2H), 7.52-7.50 (m, 4H), 7.22-6.76 (m, 6H), 6.61 (dd, J = 7.4, 2.6 Hz, 2H), 6.07 (app. brs, 1H), 5.76 (app. brs, 1H), 3.83 (s, 3H), 3.50 (app. doublet, 3H), 2.69 (app. brs, 2H), 2.56 (app. brs, 1H), 2.29 (app. brs, 1H), 1.68-1.58 (m, 4H), 1.56-1.49 (m, 2H), 1.30-1.27 (m, 2H), 1.03 (t, J = 7.2 Hz, 3H), 0.84 (app. triplet, J = 6.6 Hz, 3H); ^{13}C NMR (100 MHz, $CDCl_3$, 298 K): δ 147.8 (d, J_{PC} = 30.0 Hz), 144.5 (d, J_{PC} = 2.0 Hz), 133.9, 133.1, 131.8, 128.4, 127.5, 126.7, 126.3, 121.4, 119.8 (d, J_{PC} = 11.0 Hz), 110.4, 56.0, 55.8, 31.0, 30.9, 26.6, 26.5, 23.2, 23.0, 14.1, 13.8 (Note: The number of well-resolved ^{13}C peaks in the downfield region (> 100 ppm; 12) was less than the number of unique aromatic carbons (28) due to extensive signal broadening; ^{31}P NMR (161 MHz, $CDCl_3$, 298 K): δ 41.4, 39.1; HRMS (ESI) Calcd for $C_{40}H_{42}O_3P$ $[M + H]^+$ 601.2872, found 601.2878.

Oxidative cyclization of teraryls 7a–7c into triphenylenes 8a–8c

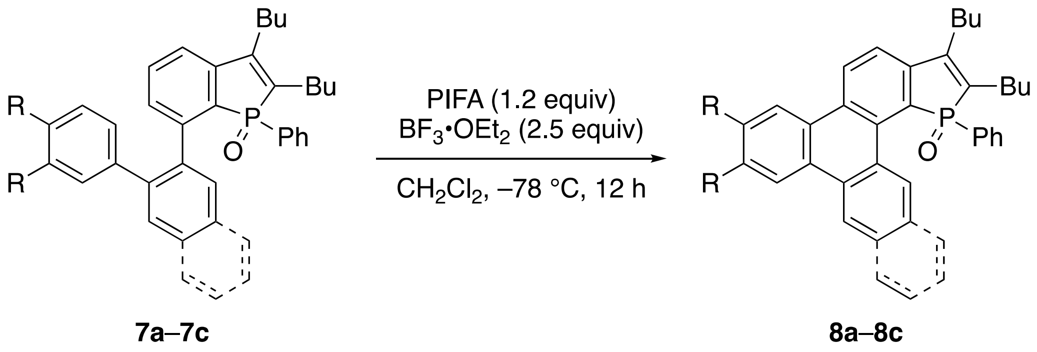

Typical procedure: To a solution of **7a** (0.1 mmol) in CH_2Cl_2 (1.0 mL) was added PIFA (0.11 mmol) and $BF_3·Et_2O$ (0.25 mmol) in a dropwise manner at −78 °C under an argon

atmosphere. The reaction mixture was stirred at −78 °C for 12 h, and then quenched with aq. NH_4Cl. The resulting mixture was extracted with EtOAc (3 × 5 mL), and the combined organic layer was dried over $MgSO_4$, and concentrated under reduced pressure. The residue was purified by silica gel chromatography (eluent: EtOAc/hexane 2:3) to afford the corresponding cyclized products.

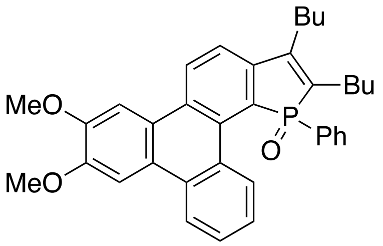

7,8-Dibutyl-2,3-dimethoxy-9-phenylphenanthro[9,10-*g*]phosphindole 9-oxide (8a): The title compound was obtained as light yellow solid (32.5 mg, 59%); m.p. 184-186 °C; ^{1}H NMR (400 MHz, $CDCl_3$, 298 K): δ 9.93-9.90 (m, 1H), 8.69 (d, J = 8.2 Hz, 1H), 8.30 (dd, J = 6.8, 2.8 Hz, 1H), 7.88 (s, 1H), 7.84 (s, 1H), 7.69 (dd, J = 8.5, 2.4 Hz, 1H), 7.58-7.52 (m, 4H), 7.30-7.27 (m, 1H), 7.20-7.15 (m, 2H), 4.09 (s, 3H), 4.06 (s, 3H), 2.73 (t, J = 7.7 Hz, 2H), 2.64-2.53 (m, 1H), 2.40-2.29 (m, 1H), 1.71-1.64 (m, 2H), 1.59-1.50 (m, 2H), 1.48-1.33 (m, 2H), 1.32-1.21 (m, 2H), 1.03 (t, J = 7.3 Hz, 3H), 0.80 (t, J = 7.2 Hz, 3H); ^{13}C NMR (100 MHz, $CDCl_3$, 298 K): δ 149.6 (d, J_{PC} = 35.5 Hz), 148.8 (d, J_{PC} = 20.0 Hz), 144.4 (d, J_{PC} = 28.9 Hz), 133.9 (d, J_{PC} = 109.2 Hz), 133.4, 131.5 (d, J_{PC} = 2.8 Hz), 131.1, 131.0, 130.7 (d, J_{PC} = 8.5 Hz), 130.3, 129.95 (d, J_{PC} = 94.1 Hz), 129.94, 128.7 (d, J_{PC} = 1.4 Hz), 128.4, 128.2, 128.1, 128.0 (d, J_{PC} = 95.7 Hz), 127.9 (2C), 126.2, 124.6, 123.9 (2C), 122.0, 120.2 (d, J_{PC} = 12.7 Hz), 104.5 (d, J_{PC} = 44.6 Hz), 56.00, 55.97, 30.9, 30.8, 26.4 (d, J_{PC} = 13.7 Hz), 26.0 (d, J_{PC} = 11.7 Hz), 23.1, 22.9, 14.0, 13.7; ^{31}P NMR (161 MHz, $CDCl_3$, 298 K): δ 45.3; HRMS (ESI) Calcd for $C_{36}H_{38}O_3P$ $[M + H]^+$ 549.2559, found 549.2562.

Recrystallization from CH_2Cl_2 afforded single crystals suitable for X-ray diffraction analysis, which unambiguously confirmed the molecular structure of **8a**.[2] The crystal packing of **8a** demonstrated the lack of π–π stacking between the triphenylene rings (Figure S1).

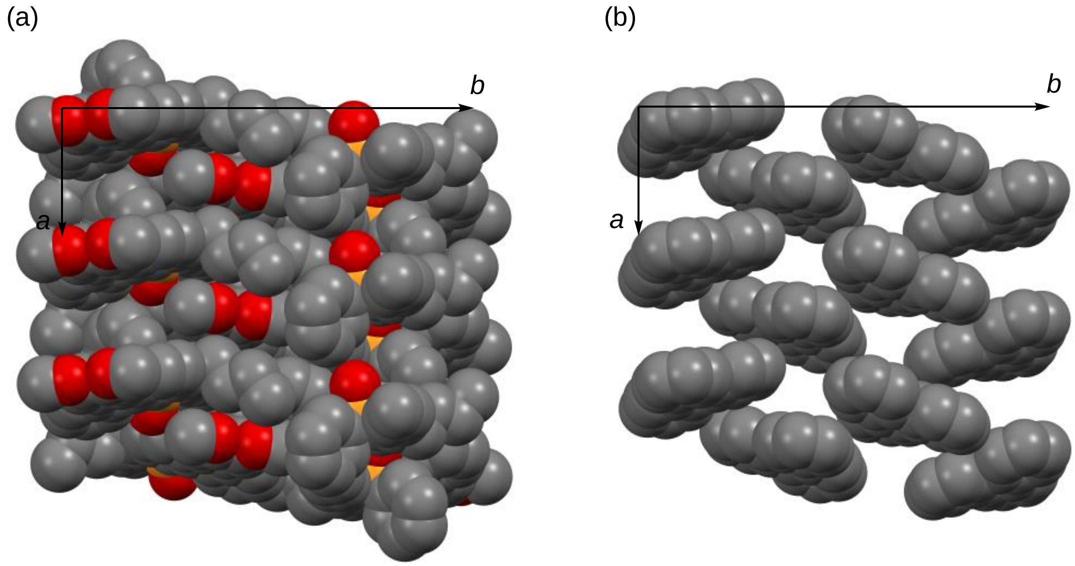

Figure S1: Crystal packing of compound **8a** showing (a) all the atoms except hydrogen and (b) only the triphenylene moieties.

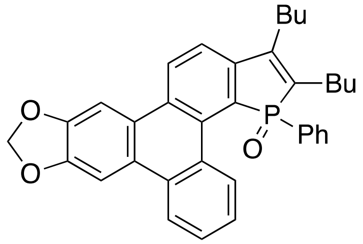

1,2-Dibutyl-3-phenylphospholo[3',2':7,8]triphenyleno[2,3-*d*][1,3]dioxole 3-oxide (8b): The title compound was obtained as light yellow solid (29.8 mg, 56%); m.p. 178-181 °C; ^{1}H NMR (400 MHz, $CDCl_3$, 298 K): δ 9.90 (d, *J* = 8.0 Hz, 1H), 8.61 (d, *J* = 8.4 Hz, 1H), 8.23 (dd, *J* = 7.0, 2.4 Hz, 1H), 7.89 (s, 1H), 7.84 (s, 1H), 7.66 (dd, *J* = 8.5, 2.3 Hz, 1H), 7.58-7.49 (m, 4H), 7.29 (dd, *J* = 7.4, 1.2 Hz, 1H), 7.20-7.15 (m, 2H), 6.07 (s, 2H), 2.72 (t, *J* = 7.6 Hz, 2H), 2.64-2.52 (m, 1H), 2.40-2.29 (m, 1H), 1.71-1.63 (m, 2H), 1.54-1.50 (m, 2H), 1.47-1.35 (m, 2H), 1.32-1.24 (m, 2H), 1.02 (t, *J* = 7.3 Hz, 3H), 0.80 (t, *J* = 7.3 Hz, 3H); ^{13}C NMR (100 MHz, $CDCl_3$, 298 K): δ 148.7 (d, J_{PC} = 19.8 Hz), 148.4 (d, J_{PC} = 23.4 Hz), 144.6 (d, J_{PC} = 29.0 Hz), 134.0 (d, J_{PC} = 102.2 Hz), 133.3 (d, J_{PC} = 7.1 Hz), 131.54, 131.51, 131.1, 131.0, 130.9, 130.8, 130.5, 129.9 (d, J_{PC} = 94.3 Hz), 129.8, 128.6 (2C), 128.4, 128.3, 128.1, 128.0 (d, J_{PC} = 95.9 Hz), 126.3, 126.2, 125.5 (d, J_{PC} = 1.5 Hz), 122.2, 120.2 (d, J_{PC} = 12.8 Hz), 102.1, 101.7 (d, J_{PC} = 23.1 Hz), 30.9 (d, J_{PC} = 1.4 Hz), 30.7 (d, J_{PC} = 2.2 Hz), 26.4 (d, J_{PC} = 13.5 Hz), 26.0 (d, J_{PC} = 11.4 Hz), 23.1, 22.9, 14.0,

13.7; ^{31}P NMR (161 MHz, $CDCl_3$, 298 K): δ 45.3; HRMS (ESI) Calcd for $C_{35}H_{34}O_3P$ $[M + H]^+$ 533.2246, found 533.2249.

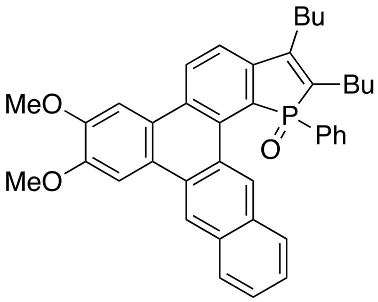

2,3-Dibutyl-7,8-dimethoxy-1-phenyltetrapheno[5,6-*g*]phosphindole 1-oxide (8c): The title compound was obtained as light yellow solid (23.9 mg, 40%); m.p. 192-194 °C; ^1H NMR (300 MHz, $CDCl_3$, 298 K): δ 10.51 (s, 1H), 8.65 (s, 1H), 8.57 (d, J = 8.5 Hz, 1H), 8.29 (dd, J = 6.2, 3.2 Hz, 1H), 7.96-7.93 (m, 2H), 7.79 (s, 1H), 7.67-7.50 (m, 5H), 7.18 (t, J = 7.1 Hz, 1H), 7.11-7.07 (m, 2H), 4.10 (s, 3H), 4.07 (s, 3H), 2.73 (t, J = 7.4 Hz, 2H), 2.67-2.54 (m, 1H), 2.44-2.35 (m, 1H), 1.71-1-64 (m, 2H), 1.59-1.40 (m, 4H), 1.33-1.25 (m, 2H), 1.03 (t, J = 7.2 Hz, 3H), 0.81 (t, J = 7.2 Hz, 3H); ^{13}C NMR (100 MHz, $CDCl_3$, 298 K): δ 149.8 (d, J_{PC} = 29.0 Hz), 149.2 (d, J_{PC} = 20.0 Hz), 144.4 (d, J_{PC} = 29.0 Hz), 134.4, 133.8 (d, J_{PC} = 96.0 Hz), 132.5, 131.7, 131.6 (2C), 131.3, 131.0, 130.9, 130.7 (d, J_{PC} = 102.0 Hz), 129.7, 129.3, 129.2, 128.6, 128.5 (d, J_{PC} = 97.0 Hz), 128.4, 128.3, 127.9, 127.3, 127.2 (d, J_{PC} = 2.0 Hz), 126.8, 125.6, 124.8, 123.9 (d, J_{PC} = 2.0 Hz), 120.5 (d, J_{PC} = 12.0 Hz), 120.3, 105.1 (d, J_{PC} = 27.0 Hz), 56.2, 56.1, 31.2, 30.9 (d, J_{PC} = 2.0 Hz), 26.5 (d, J_{PC} = 14.0 Hz), 26.1 (d, J_{PC} = 12.0 Hz), 23.2, 23.0, 14.1, 13.8; ^{31}P NMR (161 MHz, $CDCl_3$, 298 K): δ 46.0; HRMS (ESI) Calcd for $C_{40}H_{40}O_3P$ $[M + H]^+$ 599.2715, found 599.2721.

References

(1) Rahman, M. S.; Yoshikai, N. *Org. Lett.* **2019**, *21*, 3232-3236.

(2) CCDC 1984209 (**8a**) contains supplementary crystallographic data for this paper. These data can be obtained free of charge from The Cambridge Crystallographic Data Centre.

Copies of NMR spectra

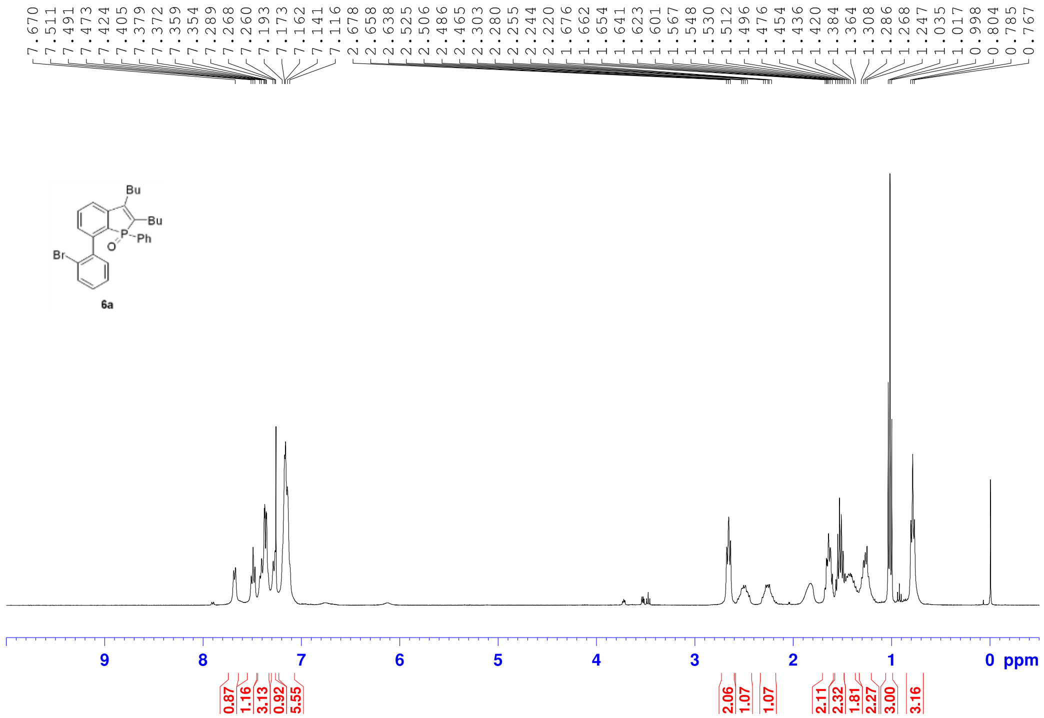

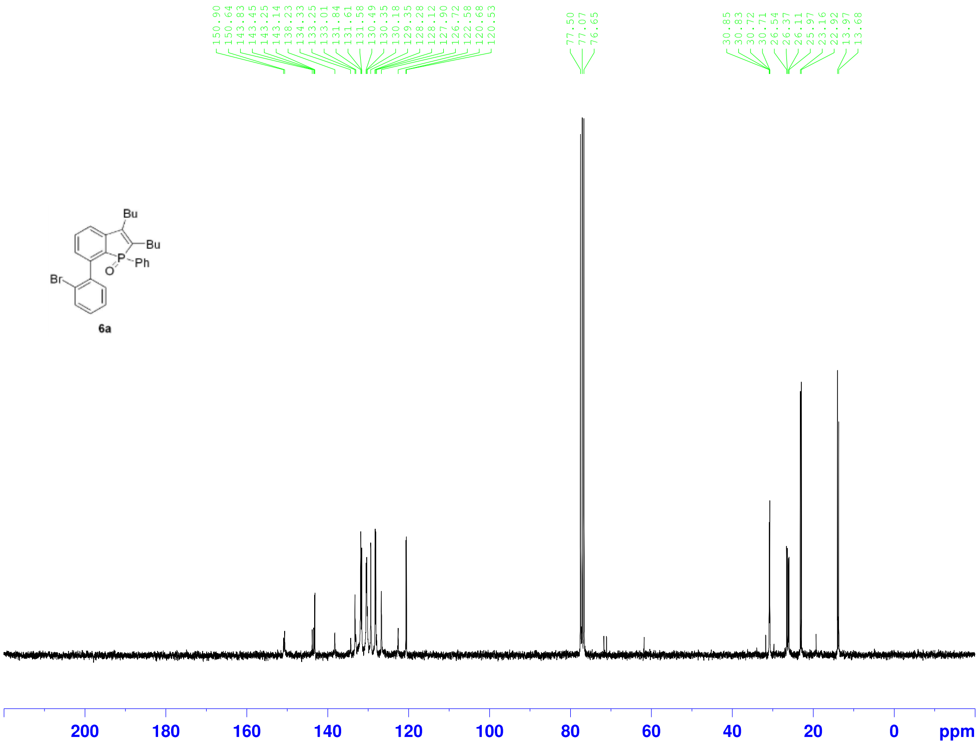
150.90
150.64
143.83
143.45
143.25
143.14
138.23
134.33
133.25
133.01
131.84
131.61
131.58
130.49
130.35
130.18
129.35
128.28
128.12
127.90
126.72
122.58
120.68
120.53
77.50
77.07
76.65
30.85
30.83
30.72
30.71
26.54
26.37
26.11
25.97
23.16
22.92
13.97
13.68
Bu
Bu
P
Ph
O
Br
6a
200
180
160
140
120
100
80
60
40
20
0
ppm

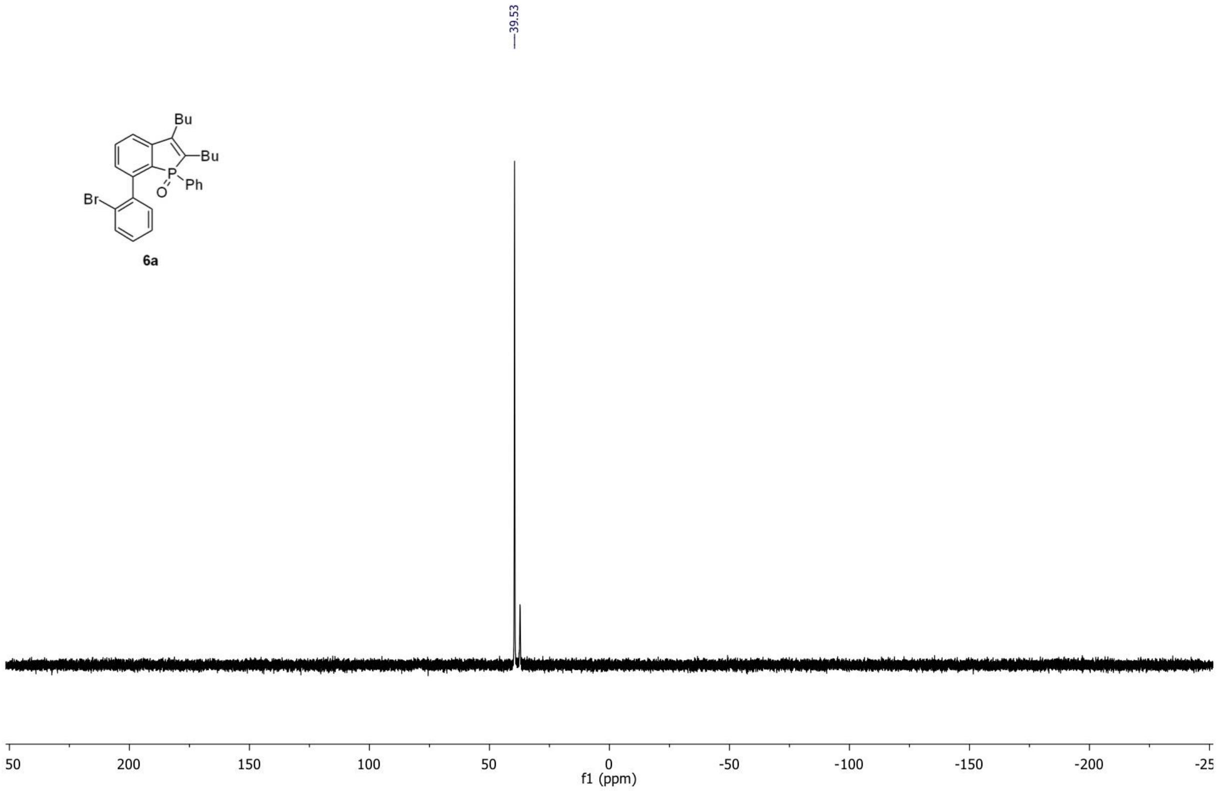

39.53
Bu
Bu
P
O
Ph
Br
6a
50
200
150
100
50
0
-50
-100
-150
-200
-25
f1 (ppm)

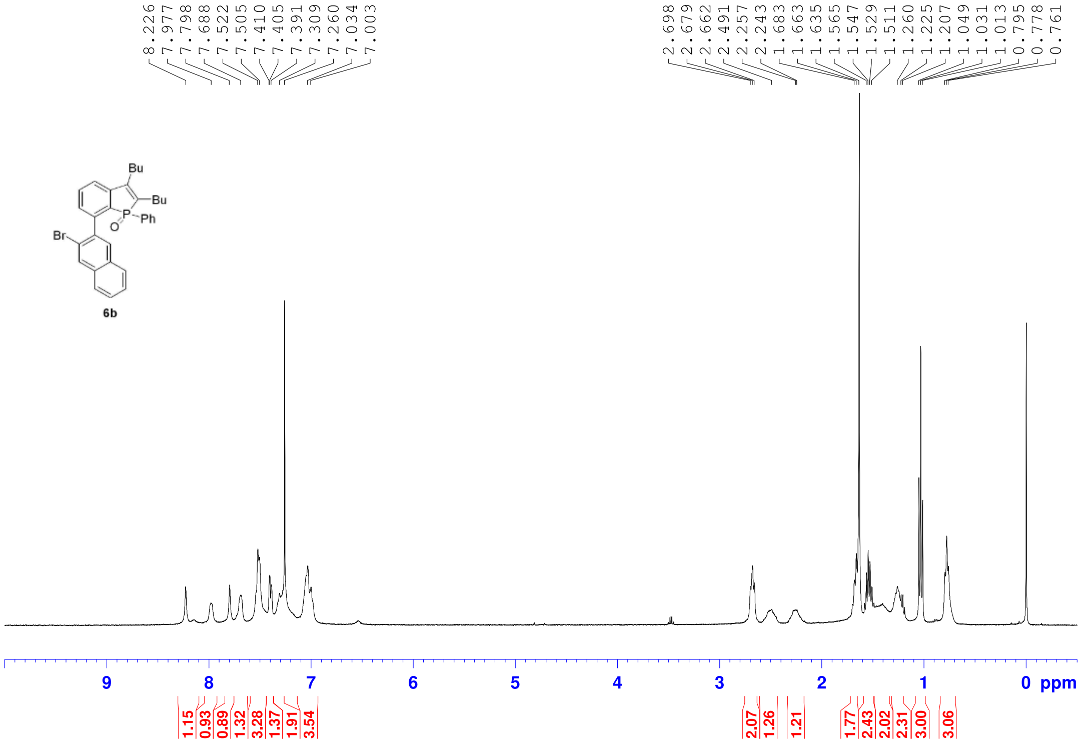
Bu
Bu
P
Ph
O
Br
8.226
7.977
7.798
7.688
7.522
7.505
7.410
7.405
7.391
7.309
7.260
7.034
7.003
2.698
2.679
2.662
2.491
2.257
2.243
1.683
1.663
1.635
1.565
1.547
1.529
1.511
1.260
1.225
1.207
1.049
1.031
1.013
0.795
0.778
0.761
9
8
7
6
5
4
3
2
1
0 ppm
1.15
0.93
0.89
1.32
3.28
1.37
1.91
3.54
2.07
1.26
1.21
1.77
2.43
2.02
2.31
3.00
3.06

6b

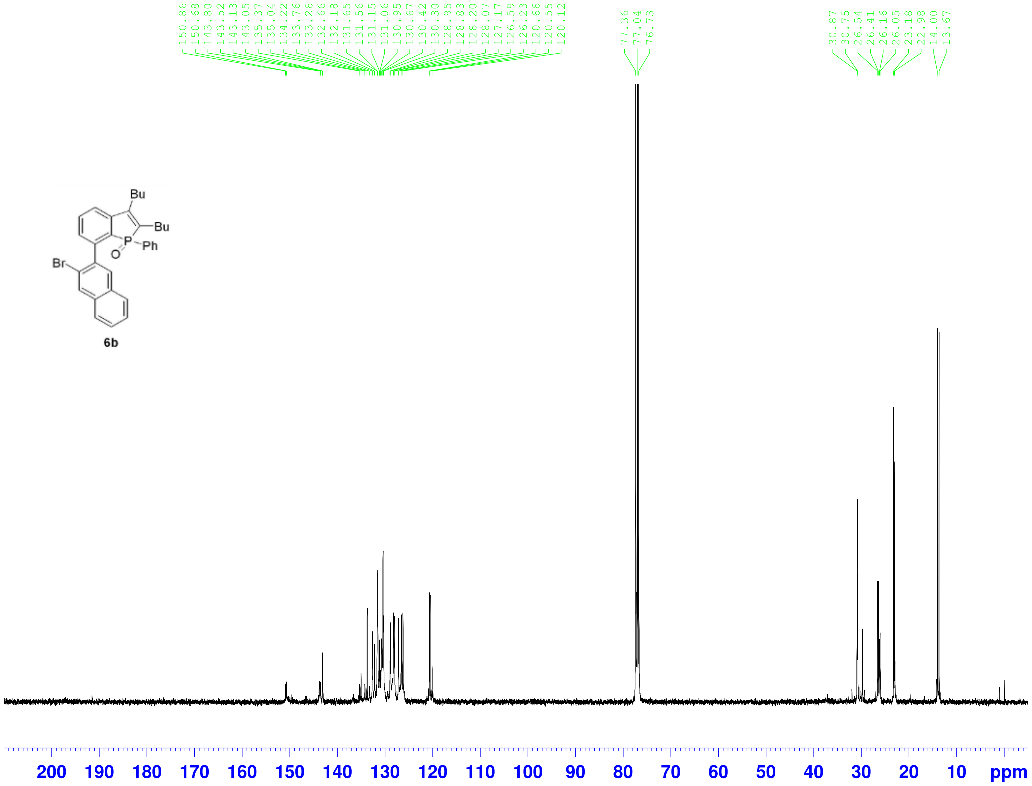
150.86
150.68
143.80
143.52
143.13
143.05
135.37
135.04
134.22
133.76
133.26
132.66
132.18
131.65
131.56
131.15
131.06
130.95
130.67
130.42
130.30
128.95
128.83
128.20
128.07
127.17
126.59
126.23
120.66
120.55
120.12
77.36
77.04
76.73
30.87
30.75
26.54
26.41
26.16
26.05
23.18
22.98
14.00
13.67
Bu
Bu
P
O
Ph
Br
6b
200 190 180 170 160 150 140 130 120 110 100 90 80 70 60 50 40 30 20 10 ppm

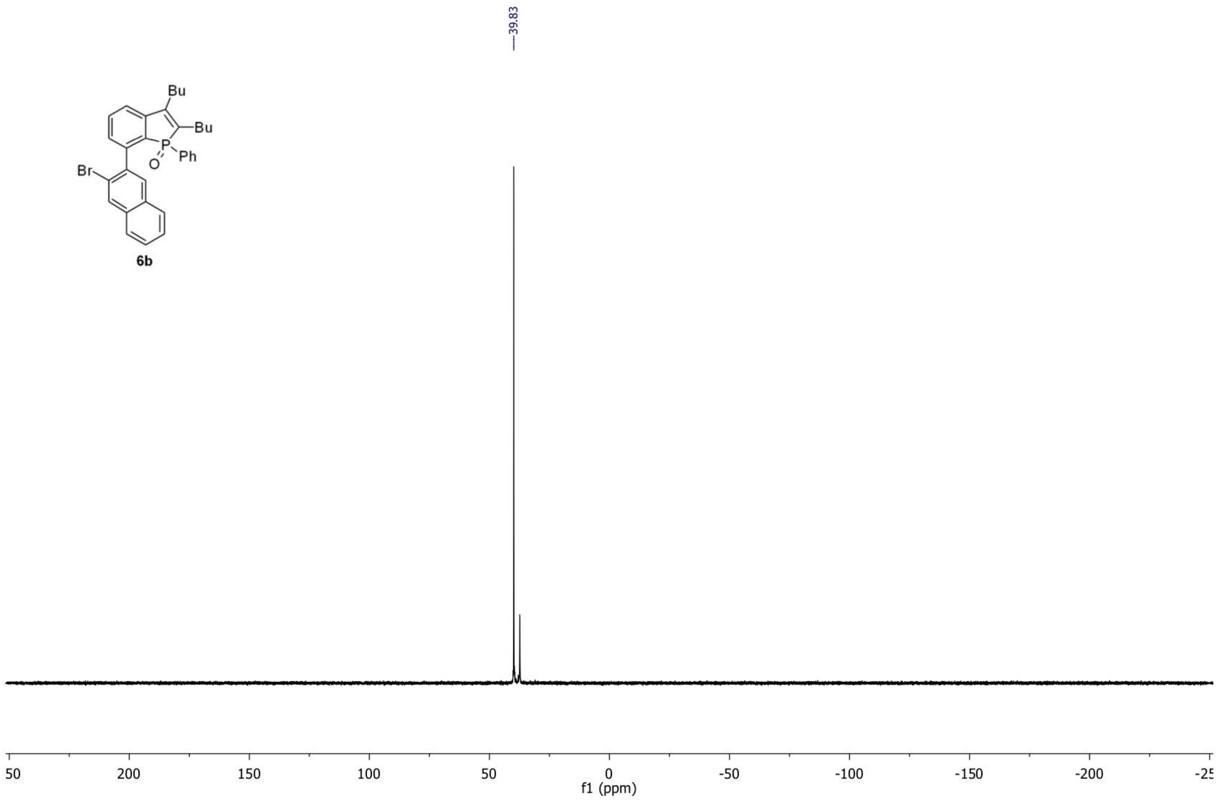
39.83
Bu
Bu
P
Ph
O
Br
6b
50
200
150
100
50
0
-50
-100
-150
-200
-25
f1 (ppm)

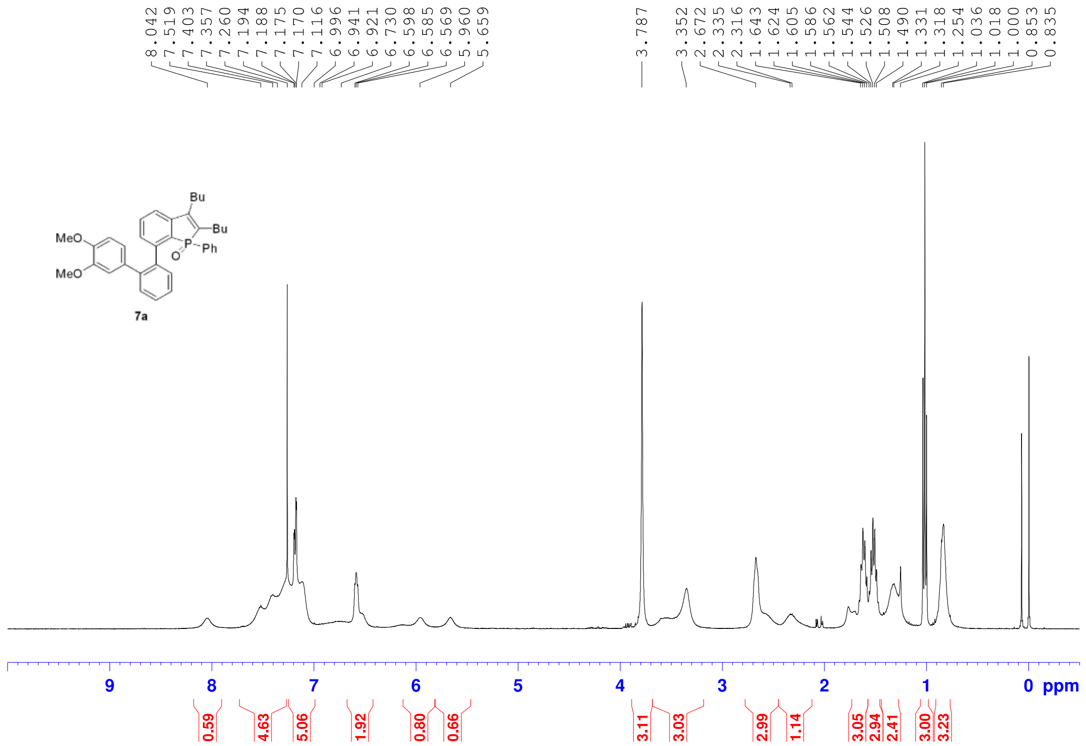
8.042 7.519 7.403 7.357 7.260 7.194 7.188 7.175 7.170 7.116 6.996 6.941 6.921 6.730 6.598 6.585 6.569 5.960 5.659
3.787
3.352 2.672 2.335 2.316 1.643 1.624 1.605 1.586 1.562 1.544 1.526 1.508 1.490 1.331 1.318 1.254 1.036 1.018 1.000 0.853 0.835
Bu
Bu
MeO
MeO
P
O
Ph
9 8 7 6 5 4 3 2 1 0 ppm
0.59 4.63 5.06 1.92 0.80 0.66 3.11 3.03 2.99 1.14 3.05 2.94 2.41 3.00 3.23

7a

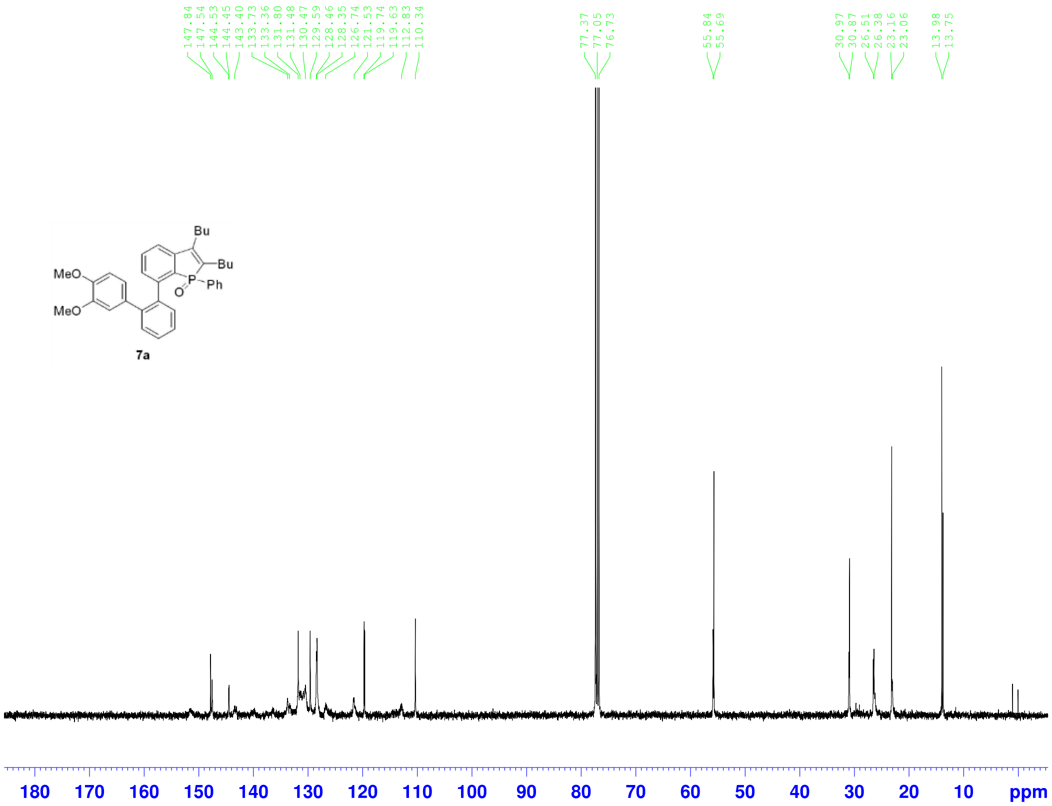
147.84
147.54
144.53
144.45
143.40
133.73
133.36
131.80
131.48
130.47
129.59
128.46
128.35
126.74
121.53
119.74
119.63
112.83
110.34
77.37
77.05
76.73
55.84
55.69
30.97
30.87
26.51
26.38
23.16
23.06
13.98
13.75
Bu
Bu
Ph
MeO
MeO
7a
180
170
160
150
140
130
120
110
100
90
80
70
60
50
40
30
20
10
ppm

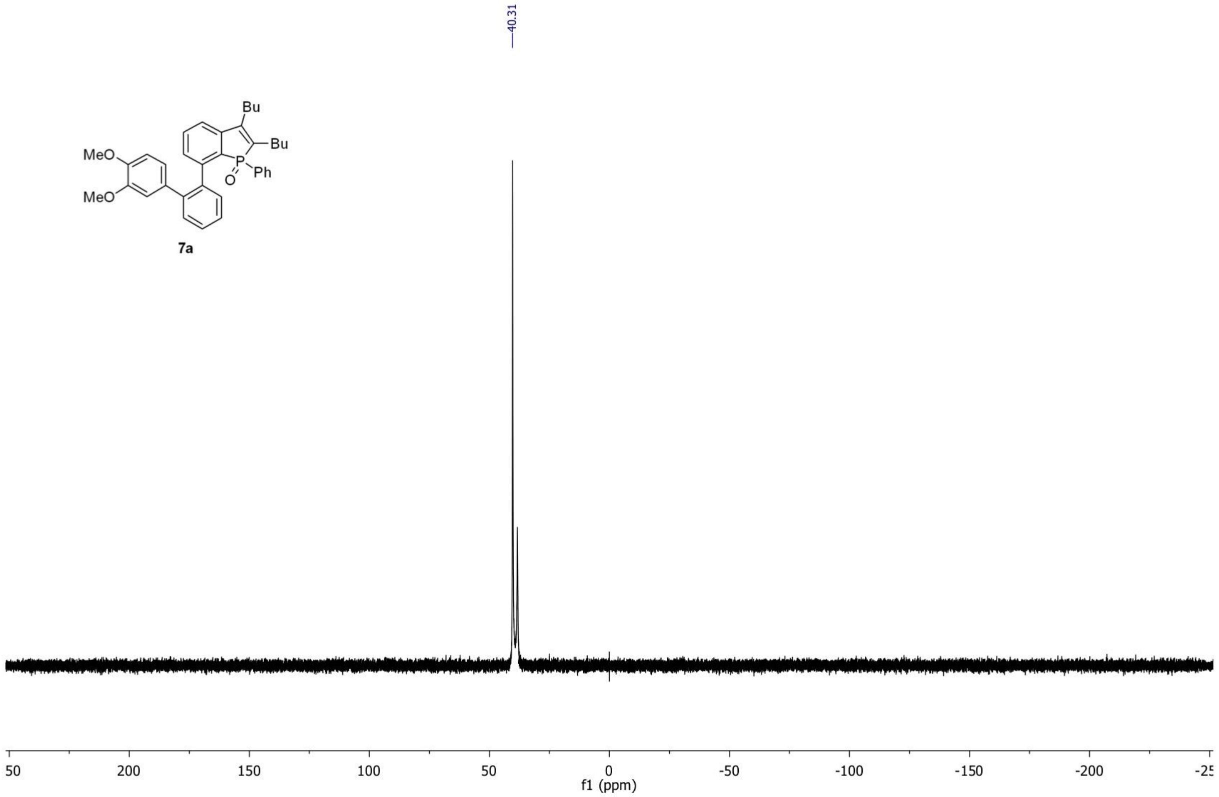
40.31
Bu
Bu
MeO
MeO
P
O
Ph
7a
50
200
150
100
50
0
-50
-100
-150
-200
-25
f1 (ppm)

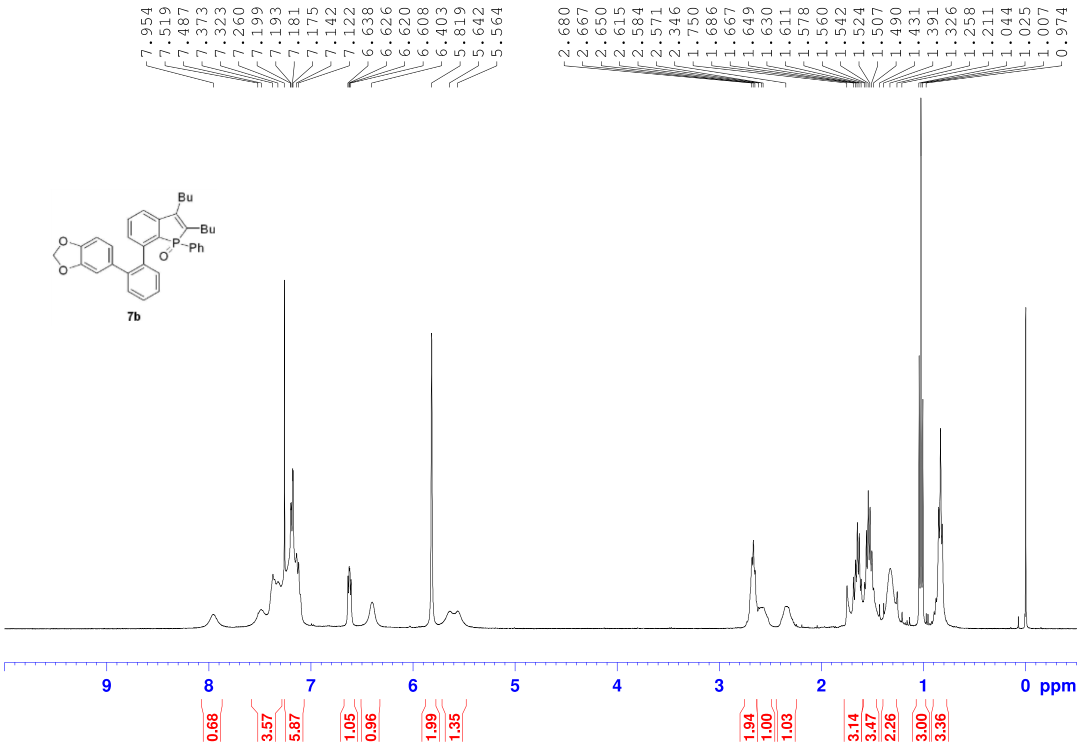

7.954
7.519
7.487
7.373
7.323
7.260
7.199
7.193
7.181
7.175
7.142
7.122
6.638
6.626
6.620
6.608
6.403
5.819
5.642
5.564
2.680
2.667
2.650
2.615
2.584
2.571
2.346
1.750
1.686
1.667
1.649
1.630
1.611
1.578
1.560
1.542
1.524
1.507
1.490
1.431
1.391
1.326
1.258
1.211
1.044
1.025
1.007
0.974
Bu
Bu
P
Ph
O
O
O
7b
9
8
7
6
5
4
3
2
1
0 ppm
0.68
3.57
5.87
1.05
0.96
1.99
1.35
1.94
1.00
1.03
3.14
3.47
2.26
3.00
3.36

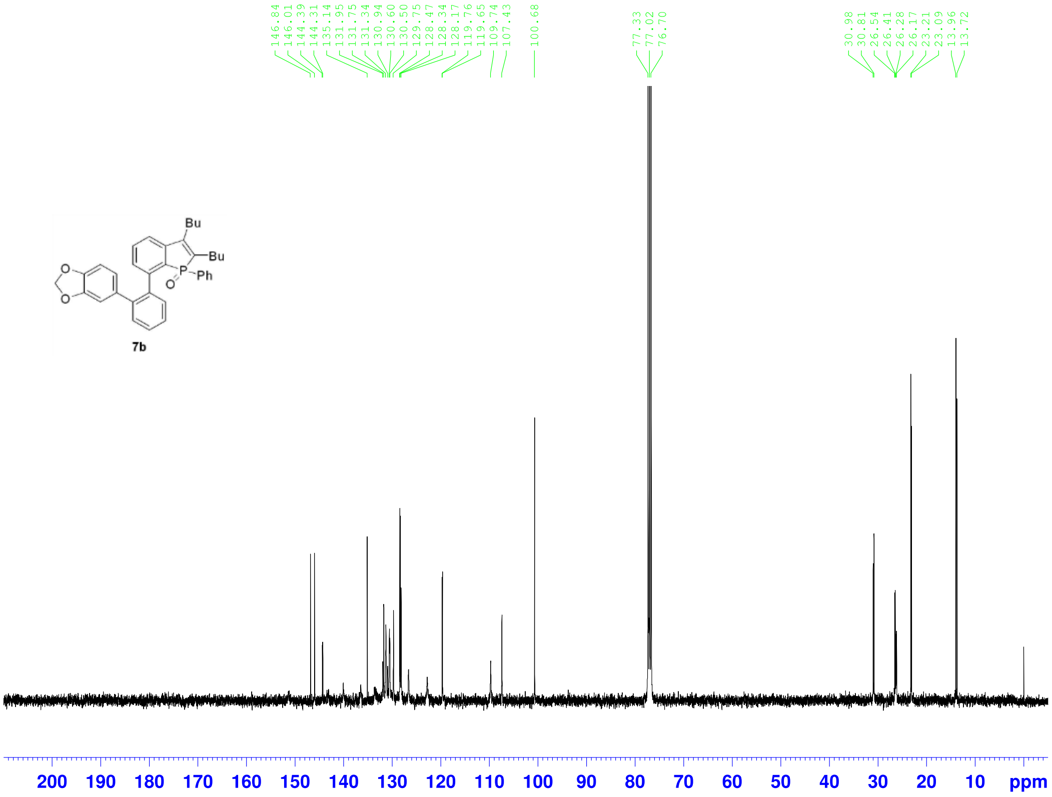
146.84
146.01
144.39
144.31
135.14
131.95
131.75
131.34
130.94
130.60
130.50
129.75
128.47
128.34
128.17
119.76
119.65
109.74
107.43
100.68
77.33
77.02
76.70
30.98
30.81
26.54
26.41
26.28
26.17
23.21
23.09
13.96
13.72
Bu
Bu
P
Ph
O
O
O
7b
200 190 180 170 160 150 140 130 120 110 100 90 80 70 60 50 40 30 20 10 ppm

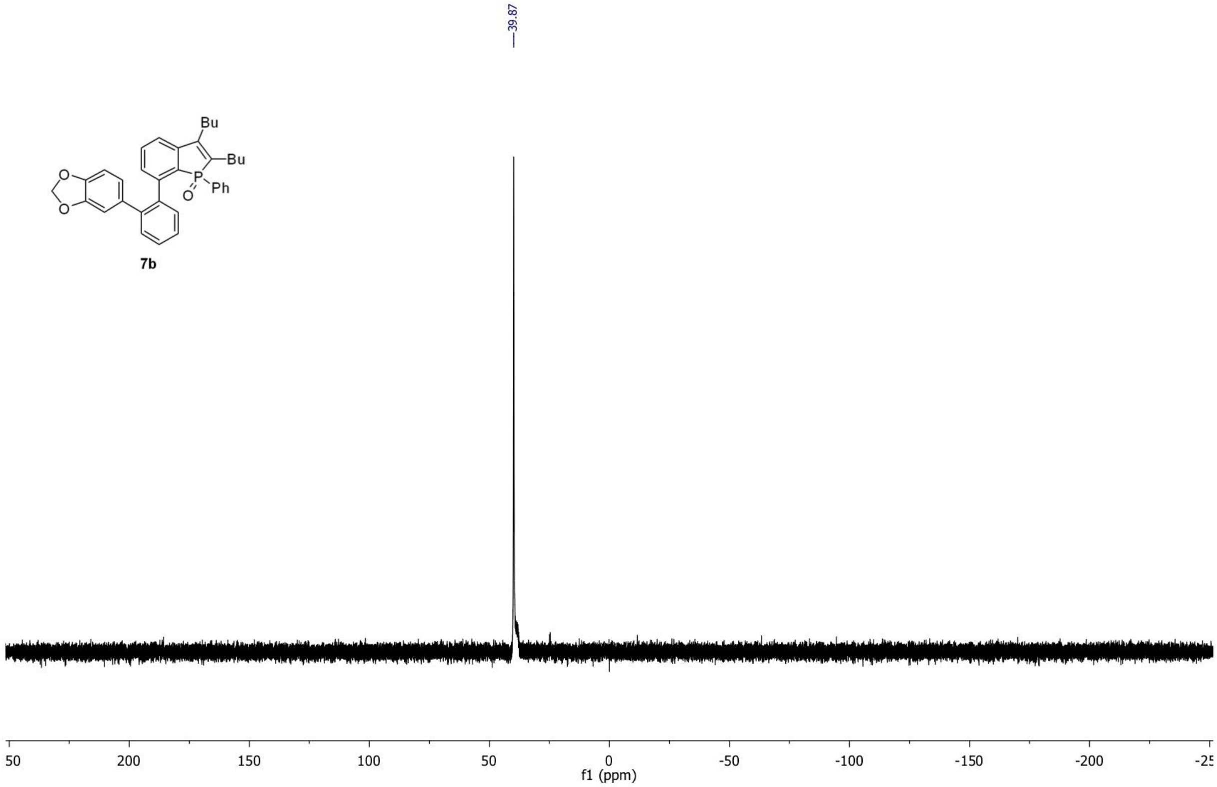

39.87
Bu
Bu
O
O
P
O
Ph
7b
50
200
150
100
50
0
-50
-100
-150
-200
f1 (ppm)

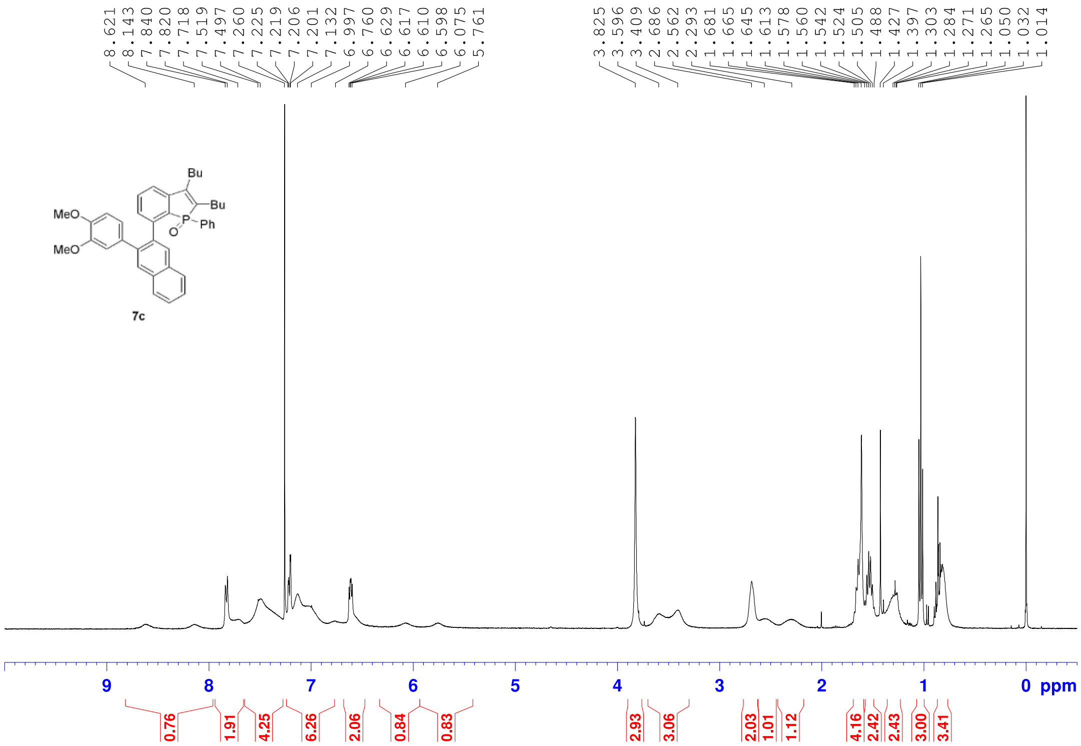
8.621
8.143
7.840
7.820
7.718
7.519
7.497
7.260
7.225
7.219
7.206
7.201
7.132
6.997
6.760
6.629
6.617
6.610
6.598
6.075
5.761
3.825
3.596
3.409
2.686
2.562
2.293
1.681
1.665
1.645
1.613
1.578
1.560
1.542
1.524
1.505
1.488
1.427
1.397
1.303
1.284
1.271
1.265
1.050
1.032
1.014
Bu
Bu
MeO
MeO
P
O
Ph
7c
9
8
7
6
5
4
3
2
1
0 ppm
0.76
1.91
4.25
6.26
2.06
0.84
0.83
2.93
3.06
2.03
1.01
1.12
4.16
2.42
2.43
3.00
3.41

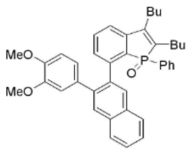

Bu
Bu
MeO
MeO
P
O
Ph

7c

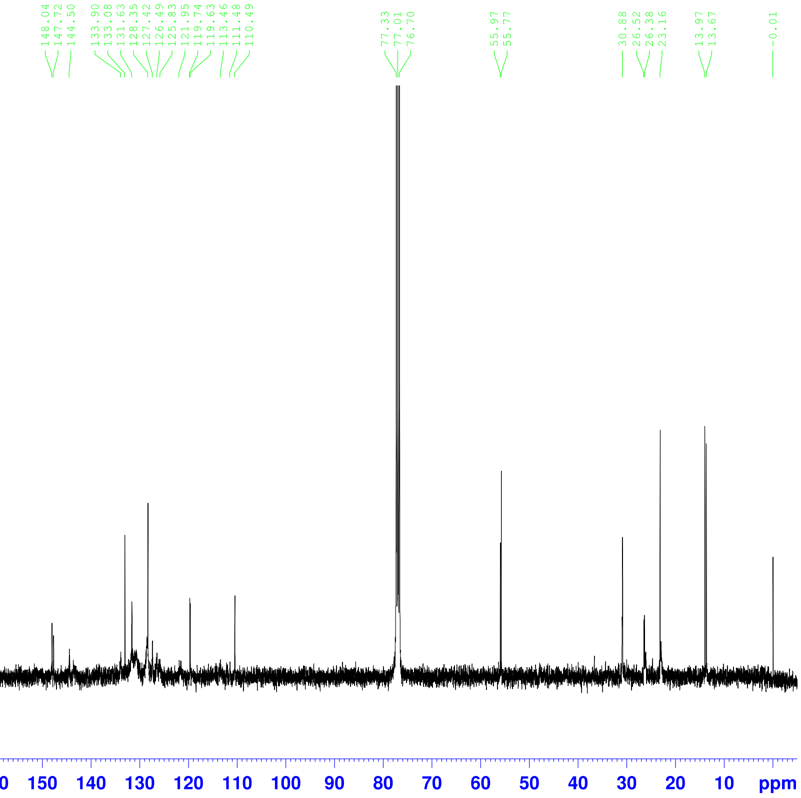

148.04
147.72
144.50
133.90
133.08
131.63
128.35
127.42
126.49
125.83
121.95
119.74
119.63
113.46
111.48
110.49
77.33
77.01
76.70
55.97
55.77
30.88
26.52
26.38
23.16
13.97
13.67
-0.01
200 190 180 170 160 150 140 130 120 110 100 90 80 70 60 50 40 30 20 10 ppm

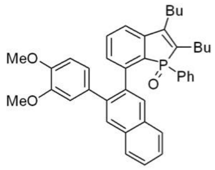
Bu
Bu
MeO
MeO
P
O
Ph

7c

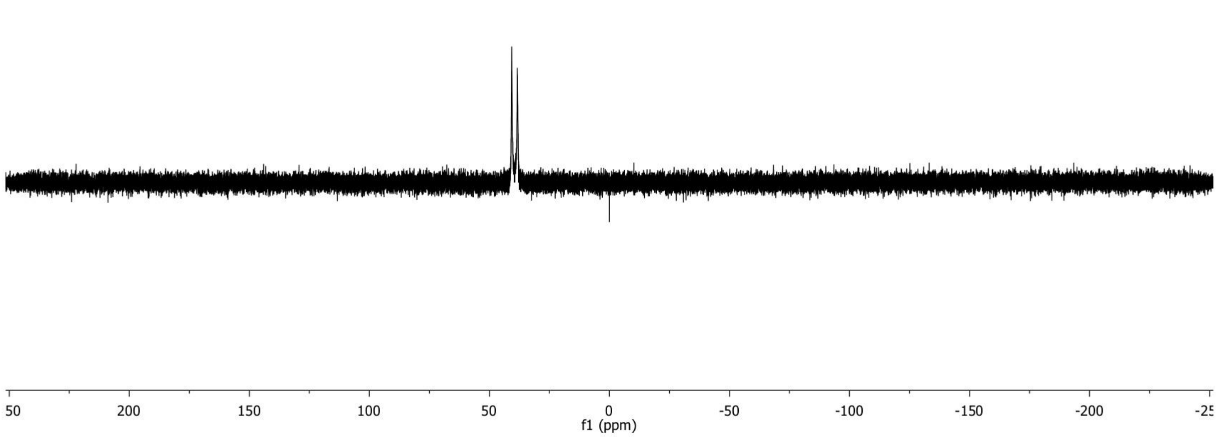
40.71
38.36
50
200
150
100
50
0
-50
-100
-150
-200
f1 (ppm)

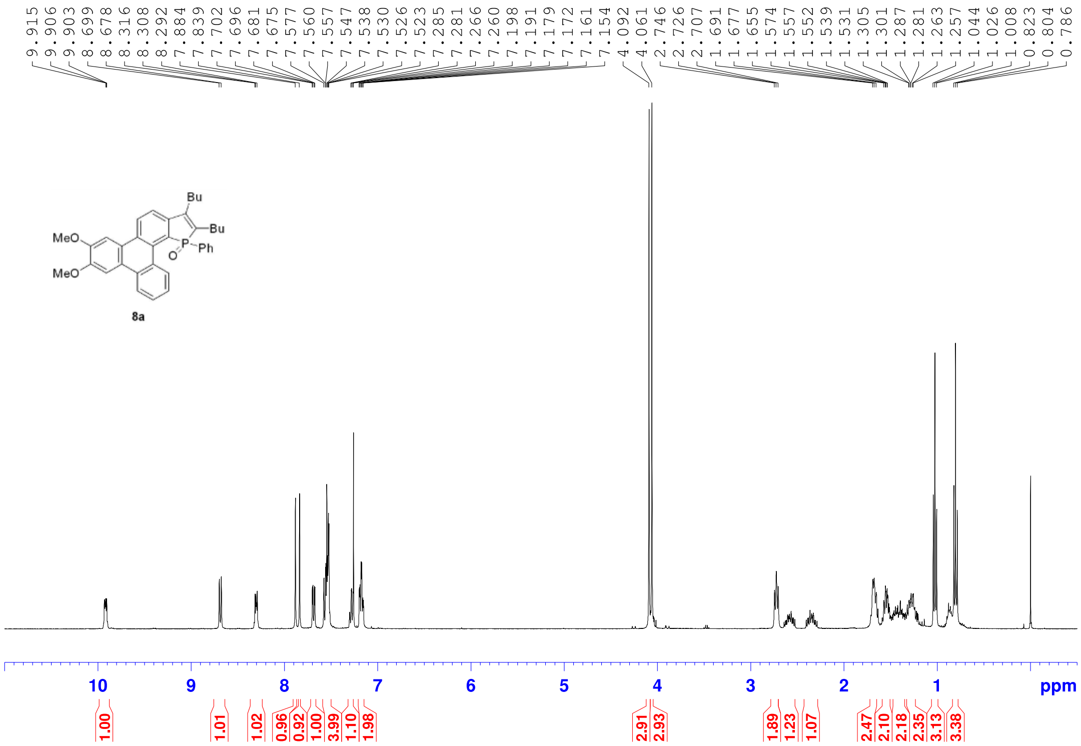
9.915
9.906
9.903
8.699
8.678
8.316
8.308
8.292
7.884
7.839
7.702
7.696
7.681
7.675
7.577
7.560
7.557
7.547
7.538
7.530
7.526
7.523
7.285
7.281
7.266
7.260
7.198
7.191
7.179
7.172
7.161
7.154
4.092
4.061
2.746
2.726
2.707
1.691
1.677
1.655
1.574
1.557
1.552
1.539
1.531
1.305
1.301
1.287
1.281
1.263
1.257
1.044
1.026
1.008
0.823
0.804
0.786
Bu
Bu
MeO
MeO
P
O
Ph
8a
10
9
8
7
6
5
4
3
2
1
ppm
1.00
1.01
1.02
0.96
0.92
1.00
3.99
1.10
1.98
2.91
2.93
1.89
1.23
1.07
2.47
2.10
2.18
2.35
3.13
3.38

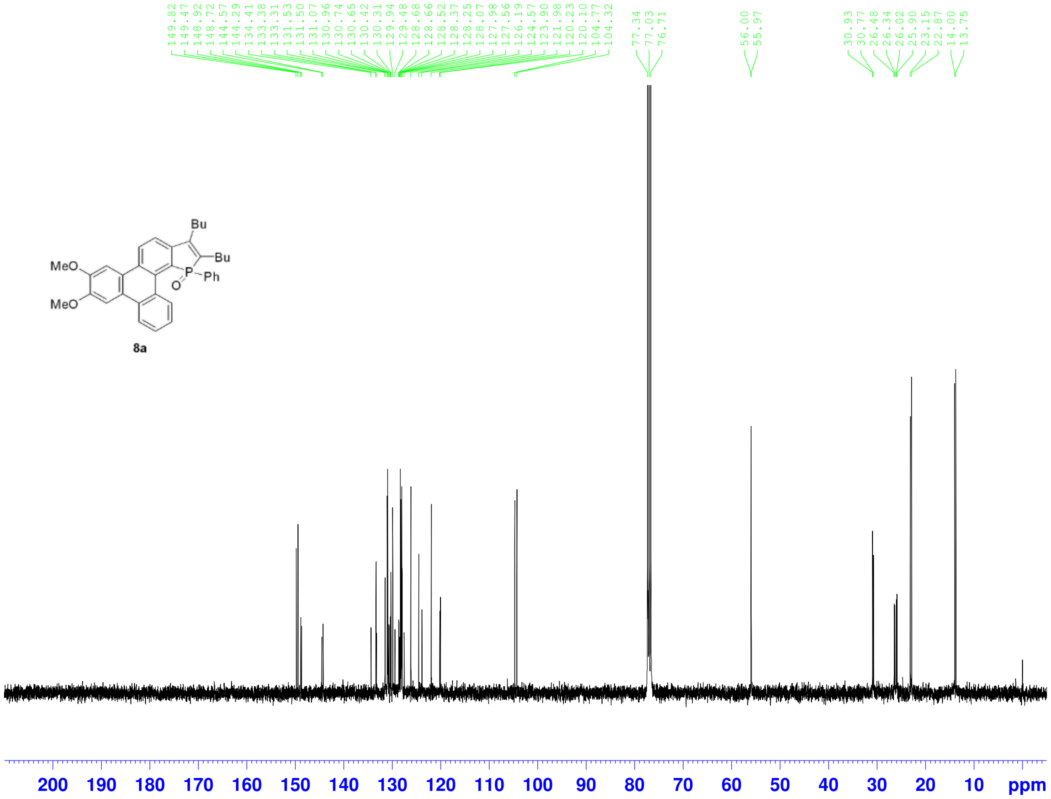
149.82
149.47
148.92
148.72
144.57
144.29
134.41
133.38
133.31
131.53
131.50
131.07
130.96
130.74
130.65
130.42
130.31
129.94
129.48
128.68
128.66
128.52
128.37
128.25
128.07
127.98
127.56
126.19
124.57
123.90
121.98
120.23
120.10
104.77
104.32
77.34
77.03
76.71
56.00
55.97
30.93
30.77
26.48
26.34
26.02
25.90
23.15
22.87
14.00
13.75
Bu
Bu
MeO
MeO
P
Ph
O
200
190
180
170
160
150
140
130
120
110
100
90
80
70
60
50
40
30
20
10
ppm
8a

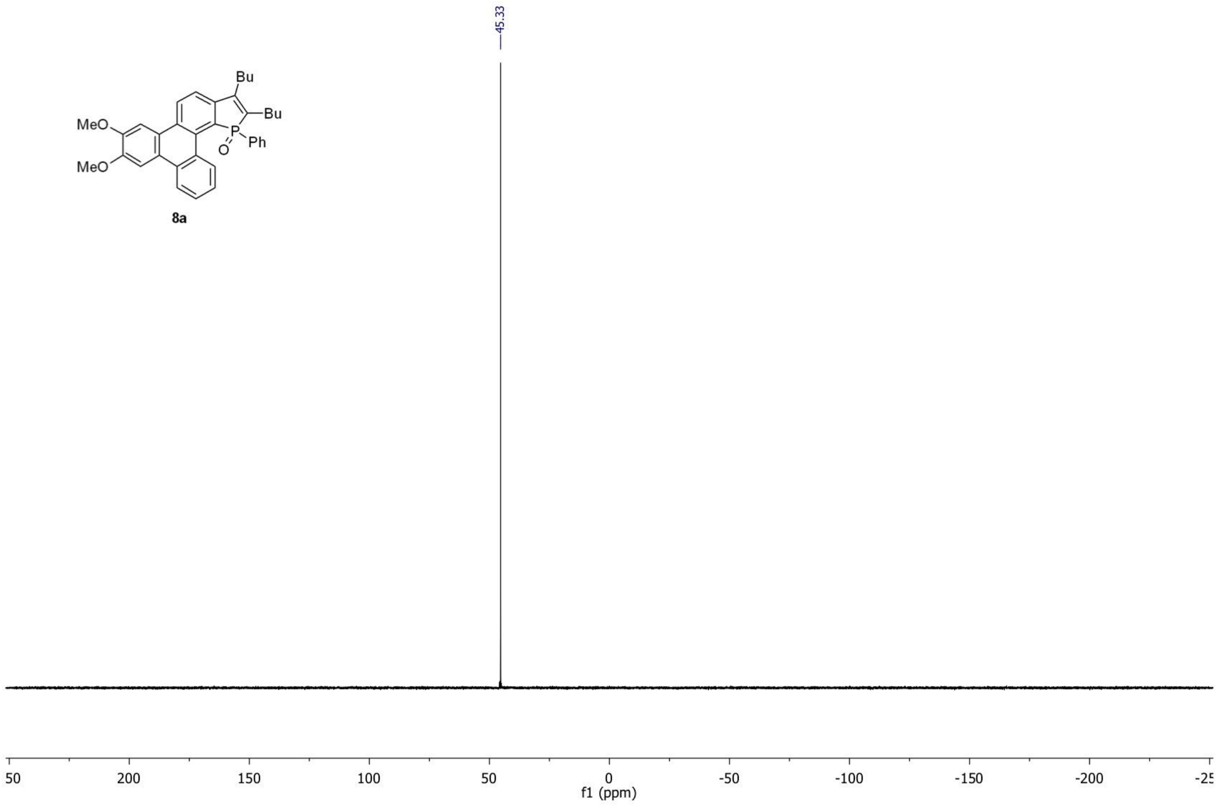

45.33
Bu
Bu
MeO
MeO
P
O
Ph
8a
50
200
150
100
50
0
-50
-100
-150
-200
-25
f1 (ppm)

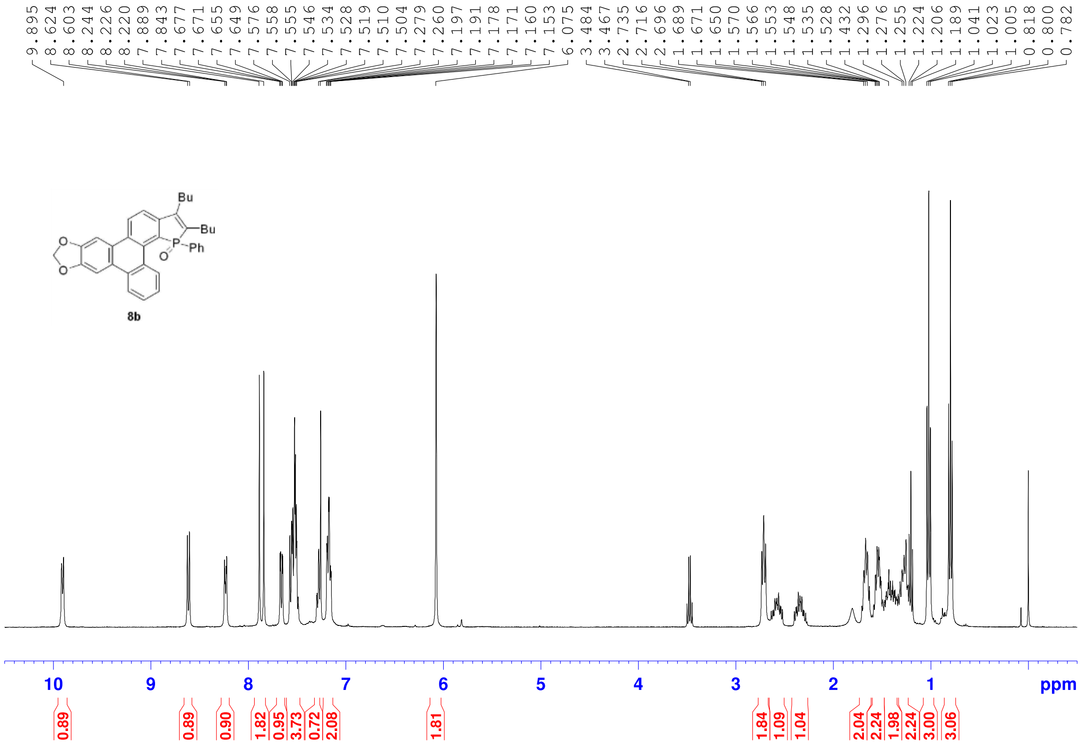
9.895 8.624 8.603 8.244 8.226 8.220 7.889 7.843 7.677 7.671 7.655 7.649 7.576 7.558 7.555 7.546 7.534 7.528 7.519 7.510 7.504 7.279 7.260 7.197 7.191 7.178 7.171 7.160 7.153 6.075 3.484 3.467 2.735 2.716 2.696 1.689 1.671 1.650 1.570 1.566 1.553 1.548 1.535 1.528 1.432 1.296 1.276 1.255 1.224 1.206 1.189 1.041 1.023 1.005 0.818 0.800 0.782
Bu
Bu
P
Ph
O
O
O
8b
10 9 8 7 6 5 4 3 2 1 ppm
0.89 0.89 0.90 1.82 0.95 3.73 0.72 2.08 1.81 1.84 1.09 1.04 2.04 2.24 1.98 2.24 3.00 3.06

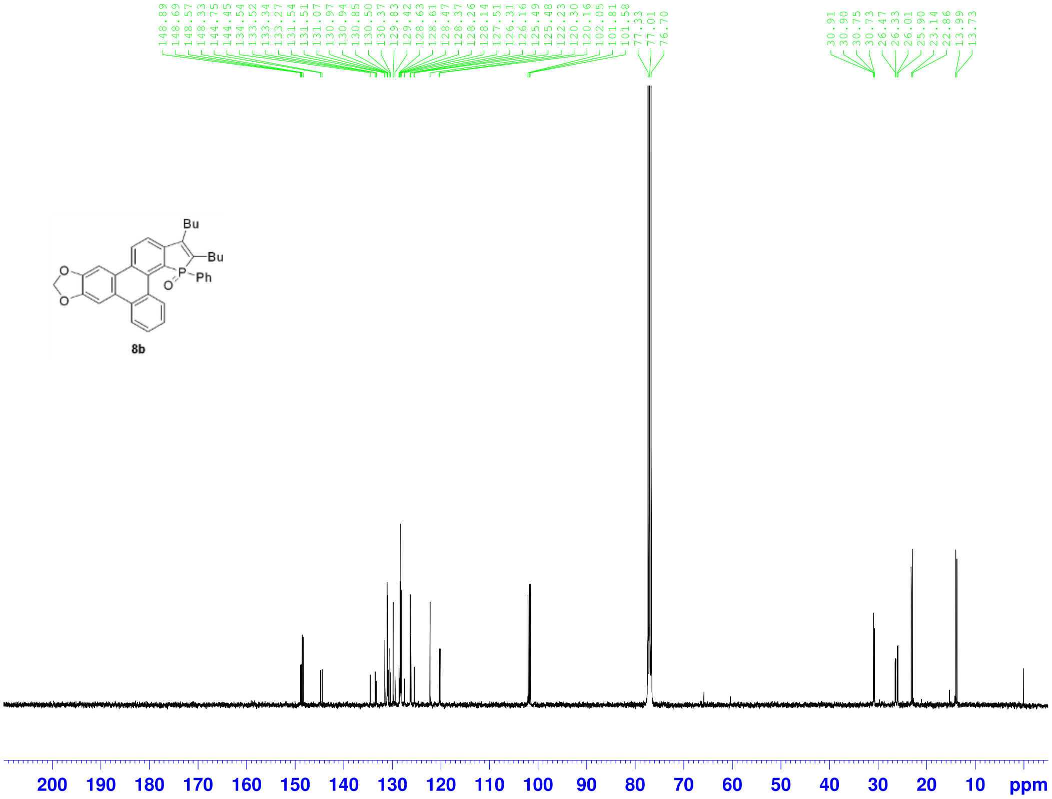
148.89 148.69 148.57 148.33 144.75 144.45 134.54 133.52 133.34 133.27 131.54 131.51 131.07 130.97 130.94 130.85 130.50 130.37 129.83 129.42 128.63 128.61 128.47 128.37 128.26 128.14 127.51 126.31 126.16 125.49 125.48 122.23 120.30 120.16 102.05 101.81 101.58 77.33 77.01 76.70
30.91 30.90 30.75 30.73 26.47 26.33 26.01 25.90 23.14 22.86 13.99 13.73
200 190 180 170 160 150 140 130 120 110 100 90 80 70 60 50 40 30 20 10 ppm
Bu
Bu
Ph

8b

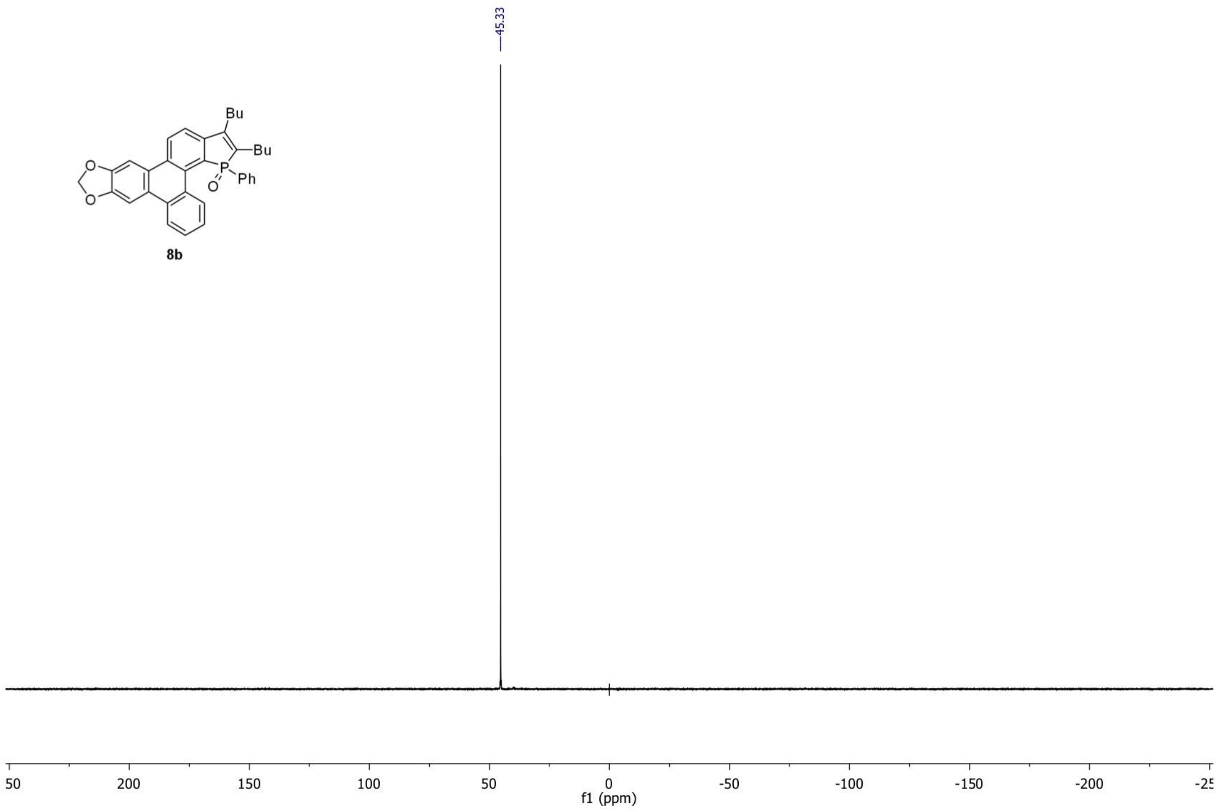
45.33
Bu
Bu
P
O
Ph
O
O
8b
50
200
150
100
50
0
-50
-100
-150
-200
-25
f1 (ppm)

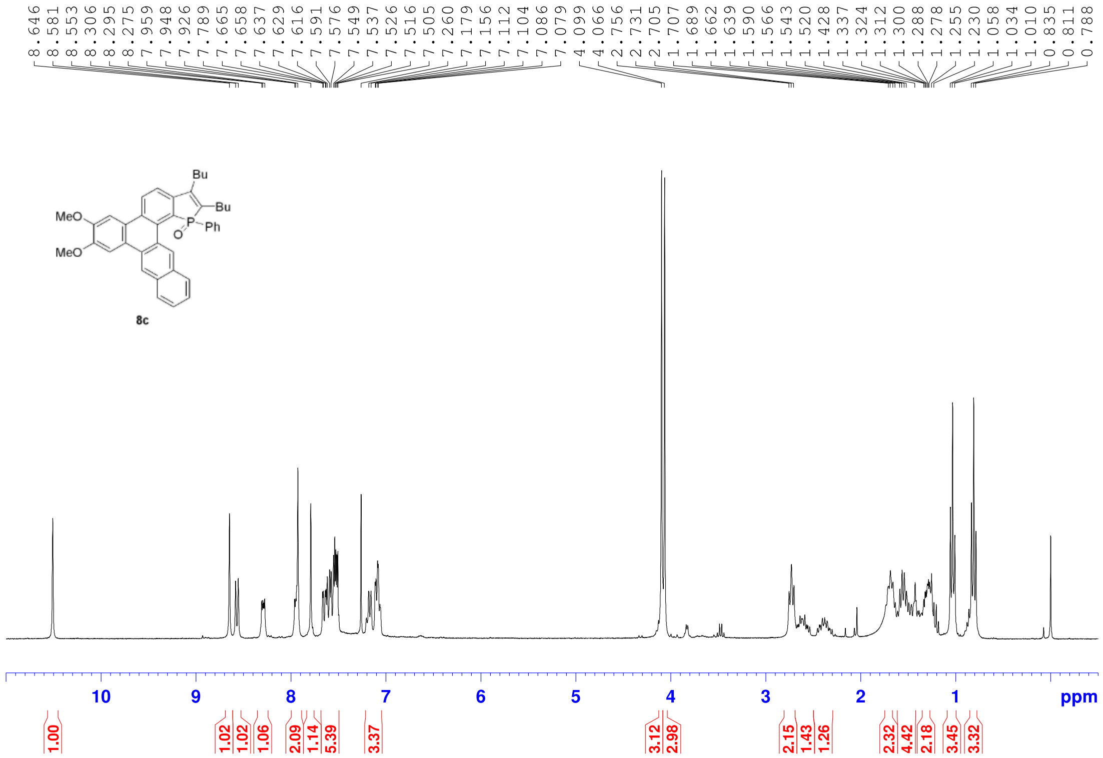
8.646
8.581
8.553
8.306
8.295
7.959
7.948
7.926
7.789
7.665
7.658
7.637
7.629
7.616
7.591
7.576
7.549
7.537
7.526
7.516
7.505
7.260
7.179
7.156
7.112
7.104
7.086
7.079
4.099
4.066
2.756
2.731
2.705
1.707
1.689
1.662
1.639
1.590
1.566
1.543
1.520
1.428
1.337
1.324
1.312
1.300
1.288
1.278
1.255
1.230
1.058
1.034
1.010
0.835
0.811
0.788
Bu
Bu
MeO
MeO
P
O
Ph
8c
10
9
8
7
6
5
4
3
2
1
ppm
1.00
1.02
1.02
1.06
2.09
1.14
5.39
3.37
3.12
2.98
2.15
1.43
1.26
2.32
4.42
2.18
3.45
3.32

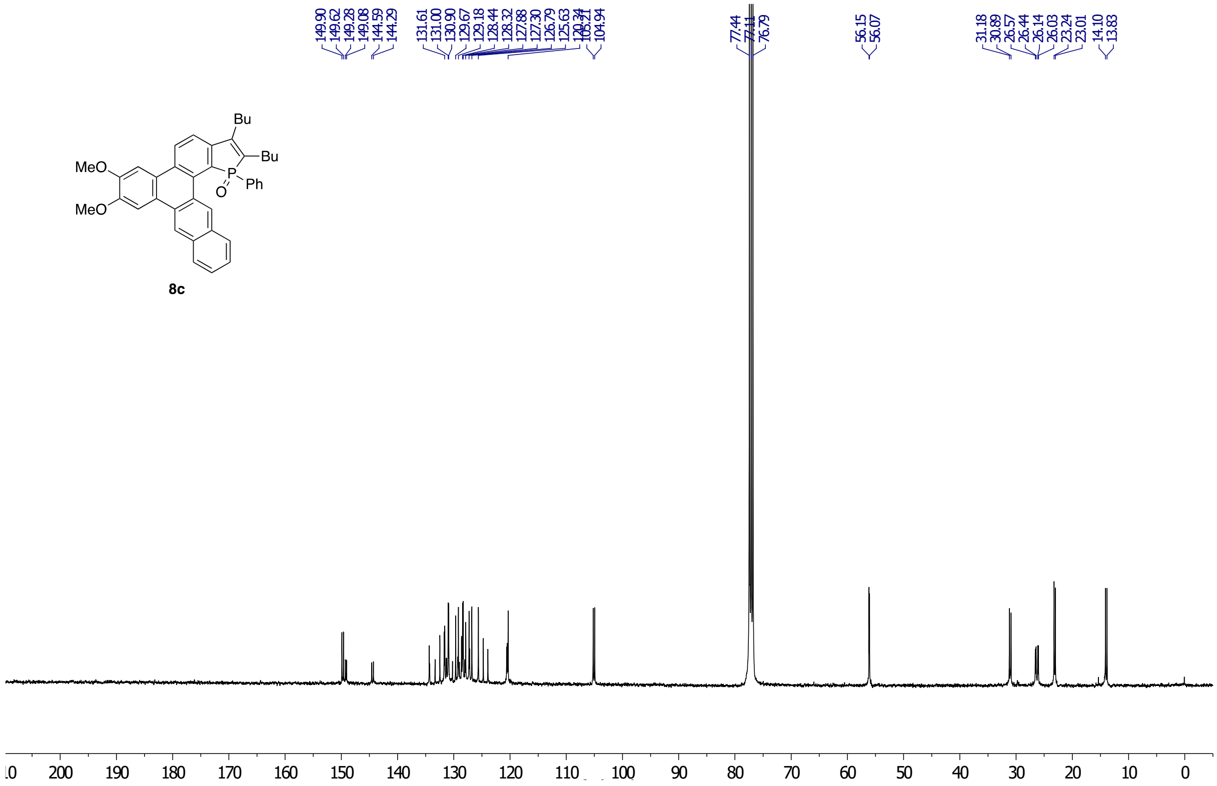
Bu
Bu
MeO
MeO
P
O
Ph
8c
149.90
149.62
149.28
149.08
144.59
144.29
131.61
131.00
130.90
129.67
129.18
128.44
128.32
127.88
127.30
126.79
125.63
104.94
77.44
77.11
76.79
56.15
56.07
31.18
30.89
26.57
26.44
26.14
26.03
23.24
23.01
14.10
13.83
200
190
180
170
160
150
140
130
120
110
100
90
80
70
60
50
40
30
20
10
0

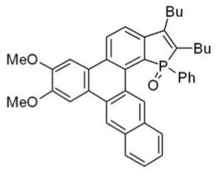

8c

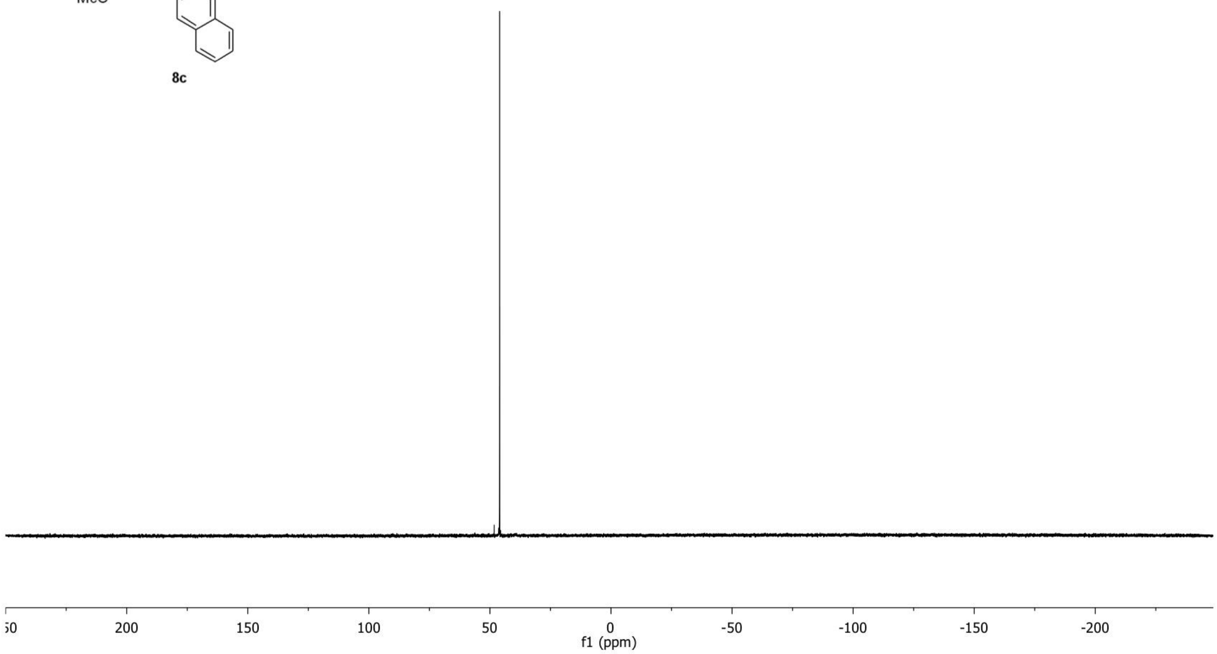

Supplement: File 1 — Experimental details and characterization data of new compounds. [file Beilstein_J_Org_Chem-16-524-s001.pdf]
